# Castaneroxy A From the Leaves of *Castanea sativa* Inhibits Virulence in *Staphylococcus aureus*

**DOI:** 10.3389/fphar.2021.640179

**Published:** 2021-06-28

**Authors:** Akram M. Salam, Gina Porras, Young-Saeng K. Cho, Morgan M. Brown, Caitlin J. Risener, Lewis Marquez, James T. Lyles, John Bacsa, Alexander R. Horswill, Cassandra L. Quave

**Affiliations:** ^1^ Program in Molecular and Systems Pharmacology, Laney Graduate School, Emory University, Atlanta, GA, United States; ^2^ Center for the Study of Human Health, Emory University, Atlanta, GA, United States; ^3^ Department of Immunology and Microbiology, University of Colorado Anschutz Medical Campus, Aurora, CO, United States; ^4^ Department of Chemistry, Emory University, Atlanta, GA, United States; ^5^ Department of Dermatology, Emory University School of Medicine, Atlanta, GA, United States; ^6^ Antibiotic Resistance Center, Emory University, Atlanta, GA, United States

**Keywords:** *Staphylococcus aureus*, MRSA, virulence, quorum sensing, AGR, natural products, ethnobotany

## Abstract

Methicillin-resistant *Staphylococcus aureus* (MRSA) represents one of the most serious infectious disease concerns worldwide, with the CDC labeling it a “serious threat” in 2019. The current arsenal of antibiotics works by targeting bacterial growth and survival, which exerts great selective pressure for the development of resistance. The development of novel anti-infectives that inhibit quorum sensing and thus virulence in MRSA has been recurrently proposed as a promising therapeutic approach. In a follow-up of a study examining the MRSA quorum sensing inhibitory activity of extracts of Italian plants used in local traditional medicine, 224C-F2 was reported as a bioactive fraction of a *Castanea sativa* (European chestnut) leaf extract. The fraction demonstrated high activity *in vitro* and effective attenuation of MRSA pathogenicity in a mouse model of skin infection. Through further bioassay-guided fractionation using reverse-phase high performance liquid chromatography, a novel hydroperoxy cycloartane triterpenoid, castaneroxy A (**1**), was isolated. Its structure was established by nuclear magnetic resonance, mass spectrometry and X-ray diffraction analyses. Isomers of **1** were also detected in an adjacent fraction. In a series of assays assessing inhibition of markers of MRSA virulence, **1** exerted activities in the low micromolar range. It inhibited *agr*::P3 activation (IC_50_ = 31.72 µM), δ-toxin production (IC_50_ = 31.72 µM in NRS385), supernatant cytotoxicity to HaCaT human keratinocytes (IC_50_ = 7.93 µM in NRS385), and rabbit erythrocyte hemolytic activity (IC_50_ = 7.93 µM in LAC). Compound **1** did not inhibit biofilm production, and at high concentrations it exerted cytotoxicity against human keratinocytes greater than that of 224C-F2. Finally, **1** reduced dermonecrosis in a murine model of MRSA infection. The results establish **1** as a promising antivirulence candidate for development against MRSA.

## Introduction

*Staphylococcus aureus*, including methicillin-resistant *S. aureus* (MRSA), remains a pathogen of great concern in the United States and across the world. In 2019, the Centers for Disease Control and Prevention (CDC) labeled *S. aureus* as a “serious threat,” and in 2017 alone *S. aureus* caused 323,700 hospitalizations, 10,600 deaths, and $1.7 billion in healthcare costs (CDC, 2019). The World Health Organization (WHO) has designated *S. aureus*, including MRSA, as a leading international pathogen of concern both in the community and in hospitals, and in 2017 it cited *S. aureus* a high priority pathogen for which new antibiotics are urgently needed (WHO, 2017; WHO, 2020). Both organizations cite antibiotic resistance in *S. aureus* as one of its most threatening characteristics. In the face of spreading antibiotic resistance, the world is in need of new anti-infective drugs (Rello et al., 2019). All currently approved antibiotic treatments exert their activity via bacteriostatic or bactericidal mechanisms (Tyers and Wright, 2019). While many efforts are being made to discover and develop antibiotics that target new pathways, much research has also been devoted to targeting bacterial virulence as an alternative approach (Alford et al., 2019; Fleitas Martínez et al., 2019). Indeed, the inhibition of virulence has been repeatedly cited and demonstrated in the literature as a promising anti-infective strategy against *S. aureus*, including MRSA (Salam and Quave, 2018; Tan et al., 2018; Wu et al., 2019).

*S. aureus* is a gram-positive bacterium that is capable of infecting numerous tissue types and has demonstrated a rapid ability to develop antibiotic resistance (Lowy, 1998; Chambers and Deleo, 2009). In order to successfully infect a host, *S. aureus* depends on a variety of virulence mechanisms largely controlled by the Accessory Gene Regulator (*agr*) quorum sensing system (Novick and Geisinger, 2008; Dayan et al., 2016). In the establishment of infection, capsular polysaccharides on the cell surface enhance tissue colonization and protect from phagocytosis (O'riordan and Lee, 2004) while cell wall-anchored proteins play roles in nutrient uptake, biofilm formation, immune evasion, and tissue adhesion and invasion (Foster et al., 2014). Later in the infection cycle, during stationary phase growth, exotoxins such as α-toxin and δ-toxin target host cells for destruction to aid in immunity disruption, nutrient acquisition, and dissemination (Fraser et al., 2000; Lin and Peterson, 2010; Otto, 2014b; Dayan et al., 2016). Since staphylococcal virulence factors are expressed under the control of quorum sensing, chemical inhibition of quorum sensing can inhibit virulence. Quorum sensing is a system of stimuli and response between cells in a bacterial population that coordinates the gene expression of pathways that contribute to virulence, the capacity to infect a host, in a population density-dependent fashion (Novick and Geisinger, 2008). The machinery of quorum sensing in *S. aureus* forms the *agr* system, the organism’s most well-studied virulence regulatory system (Quave and Horswill, 2014; Jenul and Horswill, 2018; Salam and Quave, 2018).

We have looked to nature as a source of potential quorum sensing inhibitory compounds. *Castanea sativa* leaves were reported in Italian traditional medicine for the preparation of a boiled leaf compress applied topically to treat pustulant wounds, rashes, and burns (Tartaglia, 2003). Further study of a *C. sativa* leaf extract for effects on *S. aureus* established its quorum sensing inhibitory activity (Quave et al., 2008; Quave et al., 2011).We subsequently reported the non-bacteriostatic/bactericidal quorum sensing inhibitory activity of an enriched *C. sativa* leaf extract, 224C-F2 (Quave et al., 2015). Extract 224C-F2 demonstrated high bioactivity against MRSA *in vitro* and no detectable resistance after 15 days of drug passaging (Quave et al., 2015). In a mouse skin infection model, co-administration of the extract with MRSA impaired pathogenesis without manifesting local or systemic toxicity.

*C. sativa*, commonly called the European chestnut or sweet chestnut, is a large deciduous tree belonging to the Fagaceae (beech) family. It is native to elevated forests from Iran to the Balkans, and its fruit, the chestnut, has been eaten by humans for millennia. *C. sativa* has been reported to be used by many communities around in the world in their traditional medicines. In the Kosovar Albanian Alps, decoctions are prepared from the fruits, and they are taken internally to treat headaches and externally to treat hemorrhoids (Mustafa et al., 2012). In the Marches region of Central-Eastern Italy, decoctions of the fruits are used as a hair wash to give light-colored hair a brown gloss (Pieroni et al., 2004), and a compress is made of the boiled fruit pulp to whiten facial skin (Pieroni et al., 2004). In parts of Tukey, chestnut flower tea is used to treat hemorrhoids (Wall et al., 2018). And going back to Pietro Andrea Mattioli’s 1554 commentary on Dioscorides’s *De materia medica*, chestnuts roasted with salt and pepper are attributed with aphrodisiac properties (Kosňovská, 2013).

The aim of the current study was to isolate antivirulence compounds against MRSA from *C. sativa* leaves that contribute to the plant’s ethnobotanical anti-infective value. The bioassay-guided fractionation of the previously reported active fraction 224C-F2 led to the isolation and structure elucidation of a novel hydroperoxy cycloartane triterpenoid, castaneroxy A (**1**). Furthermore, we characterize the antivirulence activity of **1** by probing its effects on transcriptional and translational products of the *S. aureus agr* system, which governs the organism’s virulence, and by examining its effects on MRSA pathogenesis *in vivo*.

## Materials and Methods

**Collection and processing of plant materials.** Fresh leaves of wild *Castanea sativa* Mill. (European chestnut) of the Fagaceae family were collected in the months of May-July in the years 2012–2014 in the Rionero-Alto Bradano region of the Basilicata Province in southern Italy. Standard guidelines for collection of wild specimens were followed (WHO, 2003). Collections were made on private land with the landowner’s consent. The voucher specimen for the collections of *C. sativa* (CQ-309) are viewable online on the SERNEC web portal (SERNEC, 2019); they were deposited at both the Herbarium Lucanum (HLUC) at the Universitá della Basilicata in Potenza, Italy and at the Emory University Herbarium (GEO) in Atlanta, GA, United States. Initial specimen identification was achieved using the standard Italian Flora (Pignatti, 2002), and this was confirmed at HLUC. *C. sativa* leaves were dried in the shade, ground with a blender, and vacuum sealed with silica packets before shipment to the US (under USDA permit P587-120409-008). At the laboratory, the leaf powder was further ground with a Thomas Wiley Mill at a 2 mm mesh size (Thomas Scientific).

**Extraction and isolation.** 224C-F2 was obtained as previously described (Quave et al., 2015). Briefly, ground, dried leaves of *C. sativa* were macerated in MeOH at room temperature for two successive periods of 72 h with daily agitation. Filtered extracts were concentrated *in vacuo*, lyophilized, then partitioned sequentially against hexanes then ethyl acetate. The resulting non-aqueous partitions were dried over anhydrous Na_2_SO_4_, concentrated *in vacuo*, and lyophilized before testing for activity. The ethyl acetate partition (224C) was subjected to further fractionation using a CombiFlash Rf+ (Teledyne ISCO) flash chromatography system using a RediSep Rf Gold silica column. Extract 224C was bonded to Celite 545 (Acros Organics) at a 1:4 ratio and dry-loaded via a RediSep dry load cartridge. Three mobile phases were employed: hexane (A), ethyl acetate (B), and methanol (C). The mobile phase gradient starts at 100% A (0% B), which is held for 6.3 column volumes (CV). From 6.3 CV to 25.3 CV, this is increased to 50% B, and from 25.3 CV to 63.3 CV, this is further increased to 100% B. 100% B is held until 69.9 CV. From 69.9 CV to 88.6 CV, the gradient changes to 70:30 B:C, and 30% C is held until 94.9 CV. The wavelengths monitored were 254 and 280 nm. 224C-F2c represents the portion of 224C-F2 that elutes from 28.5 CV to 37.1 CV.

Method development for the fractionation of 224C-F2c via HPLC was performed on analytical HPLC. An Agilent 1260 Infinity system running OpenLab CDS ChemStation (Agilent Technologies, Santa Clara, CA, United States) was used with an Agilent XDB-C18 (250 mm × 4.6 mm, 5 μm) column with a compatible guard column at a column temperature of 25°C. Mobile phase reagents were HPLC-grade and purchased from Fisher Scientific except for the Type 1 water, which was obtained from an EMD Millipore MILLI-Q water system (Billerica, MA). The mobile phases were 0.1% formic acid in acetonitrile (A) and 0.1% formic acid in water (B); the flow rate was 1 mL/min. Production of 224C-F2c fractions was performed on an Agilent 1260 Infinity II system running the same software. The column used was an Agilent XDB-C18 (250 mm × 30 mm, 5 μm) column. 224C-F2c was dissolved in MeOH and 2 mL injections were made. Chromatograms were monitored at 254 and 314 nm. Initial conditions were 98:2 (A:B), held for 5.5 min, changing to 43:57 (A:B) at 14.5 min and held till 23.5 min, changing to 2:98 (A:B) at 26.5 min and held until 45 min. A total of 43 “preparative fractions” (PFs) were obtained using this method. PF42 eluted from 35.0–35.5 min, and due to its activity in the *S. aureus agr* reporter strain panel, it was chosen for further fractionation.

A second round of preparative HPLC fractionation to split 224C-F2c-PF42 into “subfractions” (SFs) utilized an Agilent XDB-C18 (50 mm × 30 mm, 5 μm) column. Method development was performed on the analytical system cited above, and a custom-built open-bed fraction collector was used for preparative HPLC (Caputo et al., 2020). Initial conditions were 70:30 (A:B), changing to 43:57 (A:B) at 2.00 min and held until 6.50 min, changing to 0:100 (A:B) at 8.00 min and held until 12.50 min, and then held at 70:30 (A:B) from 12.51 to 16.00 min. 224C-F2c-PF42 was dissolved in MeOH and 1 mL injections were made. Chromatograms were monitored at 217 and 254 nm. 224C-F2C-PF42-SF6, containing compounds **2a/2b**, eluted from 2.5–3.0 min; one run yielded 1.12 mg (11.2% yield). 224C-F2c-PF42-SF7, containing compound **1** (castaneroxy A), eluted from 3.0–3.5 min; one run yielded 0.47 mg (4.7% yield). Castaneroxy A represents 0.0019% of dried *C. sativa* leaves.

Castaneroxy A (**1**): white amorphous solid; [α]^23^_D_ +51.0 (c 0.003, MeOH); UV (MeOH) λ_max_ (log *ε*) 210 (1.04) nm; IR υ_max_ 3352, 2932, 2866, 1696, 1558, 1443, 1374, 1260, 1077, 996, 925, 902, 846, 768, 750 cm^−1^; HRESIMS m**/**z 503.3381 [M–H]^-^ (calc. for 503.3367 C_30_H_47_O_6_); ^1^H and ^13^C NMR data (Table 1).

**TABLE 1:** ^1^H and ^13^C NMR data (δ, in ppm) for compounds **1** and **2a**, **2b**.

Position	1		2a (major)		2b (minor)	
	δC, type	δH, mult. (J in Hz)	δC, type	δH, mult. (J in Hz)	δC, type	δ_H_, mult. (*J* in Hz)
1a	31.9, CH_2_	1.65, m	31.9, CH_2_	1.67, m	–	–
1b	–	1.37, m	–	1.39, m		–
2a	30.2, CH_2_	1.73, m	30.2, CH_2_	1.75, m	–	–
2b	–	1.29, m	–	1.30, m		–
3	76.4, CH	4.01, m	76.2, CH	4.01, dd (11.6, 4.0)	–	–
4	55.8, C^a^	--	55.3, C	--	–	–
5	43.3, CH	2.13, m	43.4, CH	2.13, dd (13.2, 3.7)	–	–
6a	33.3, CH_2_	1.45, m	33.3, CH_2_	1.41, m	–	–
6b	–	1.11, m	–	1.14, m		–
7	70.7, CH	3.53, brs	70.5, CH	3.53, brs	–	–
8	54.5, CH	1.73, m	54.5, CH	1.74, m	–	–
9	29.3, C	--	29.2, C	--	–	–
10	21.3, C	--	21.4, C	--	–	–
11a	28.1, CH_2_	1.78, m	28.1, CH_2_	1.78, m	–	–
11b	–	1.43, m	–	1.46, m		–
12	33.9, CH_2_	1.60, m	33.6, CH_2_	1.60, m	–	–
13	46.7, C	--	46.7, C	--	–	–
14	49.9, C	--	49.8, C	--	–	–
15a	36.9, CH_2_	1.59, m	37.0, CH_2_	1.59, m	–	–
15b	–	1.46, m	–	1.47, m		–
16a	29.2, CH_2_	1.30, m	29.1, CH_2_	1.95, m	–	–
16b	–	1.30, m	–	1.33, m		
17	52.8, CH	1.56, m	52.5, CH	1.56, m	–	–
18	17.2, CH_3_	1.00, s	17.3, CH_3_	1.02, s	–	–
19a	27.1, CH_2_	0.80, d (4.5)	27.5, CH_2_	0.80, d (4.7)	–	–
19b	–	0.30, d (4.4)	–	0.30, d (4.6)		–
20	37.4, CH	1.42, m	37.7, CH	1.51, m	–	–
21	19.0, CH_3_	0.90, d (6.4)	19.0, CH_3_	0.90, d (6.4)	–	–
22a	33.7, CH_2_	1.60, m	40.5, CH_2_	2.21, m	–	–
22b	–	1.60, m	–	1.81, m	–	–
23a	28.7, CH_2_	1.89, m	129.7, CH	5.62, ddd (16.0, 7.8, 2.6)	–	–
23b	–	1.31, m	–	–	–	–
24	90.9, CH	4.16, dd (6.8, 6.8)	137.1, CH	5.57, d (16.0)	91.0, CH	4.17, dd (6.8, 6.8)
25	146.1, C	--	82.5, C	--	145.7, C	--
26a	114.0, CH_2_	4.93, dq (1.6, 1.6)	24.9, CH_3_	1.29, s	114.4, CH_2_	4.94, dq (1.6, 1.6)
26b	–	4.91, m	–	–	–	4.91, m
27	17.1, CH_3_	1.71, s	25.1, CH_3_	1.29, s	16.8, CH_3_	1.71, s
28	182.6, C*^a^*	--	180.9, C	--	–	–
29	10.3, CH_3_	1.10, s	9.8, CH_3_	1.10, s	–	–
30	19.2, CH_3_	0.95, s	19.2, CH_3_	0.94, s	–	–

a δ_C_ determined by 2D experiments.

**General Experimental Procedures.** Optical rotations were measured using a Perkin-Elmer 341 polarimeter (concentration in g/100 mL) with methanol as a solvent. IR spectra were recorded on a Nicolet iS10 FT-IR spectrometer and the absorption peaks were reported in cm^−1^. Nuclear magnetic resonance (NMR) data were recorded on a Bruker 600 Ascend (600 MHz for ^1^H NMR and 150 MHz for ^13^C NMR) instrument equipped with CryoProbe™ Prodigy. NMR spectra were recorded in solutions of deuterated methanol (MeOD) with the residual methanol (3.31 ppm for ^1^H NMR and 49.15 ppm for ^13^C NMR) taken as the internal standard; they were reported in parts per million (ppm) relative to tetramethylsilane (TMS) at 0 ppm. Mass spectrometric data was acquired in MS1 mode scanning from a *m/z* of 150–1,500 on a Thermo Scientific LTQ-FT Ultra MS in negative ESI mode and processed with Thermo Scientific Xcalibur 2.2 SP1.48 software (San Jose, CA). Samples were directly injected. The capillary temperature was 275.0°C, sheath gas of 60, source voltage and current were 5.0 kV and 100.0 μA, and the capillary voltage was −49.0 V 3D molecular modeling was performed using ChemBioDraw Ultra and Chem3D. All solvents were acquired from Fisher Chemical, Certified ACS. Target compounds and side products are labeled with Arabic numerals.

**Single Crystal X-ray Diffraction.** Colorless crystals were initially obtained by evaporating the fractions 224C-F2c-PF42-SF6 and 224C-F2c-PF42-SF7 under a barvap. Larger crystals suitable for X-ray crystallographic analysis of castaneroxy A were later obtained by slow evaporation from a chloroform-hexane mixture. Slow evaporation of a CH_3_OH solution of the mixture **2a/2b** yielded crystals of **2a** suitable for a single-crystal X-ray diffraction study. Suitable crystals were mounted on a loop with paratone. X-ray diffraction data were collected on a Rigaku XtaLAB Synergy-S diffracrometer with a HyPix-6000HE detector. The crystals were cooled to 100 K with an Oxford Cryosystems low-temperature device during the collections. Data were measured using ω scans of 0.5° per frame for variable scan times using CuK_α_ radiation (micro-focus sealed X-ray tube, 50 kV, 1.0 mA). The total number of runs and images was based on the strategy calculation from the program CrysAlisPro (Rigaku, V1.171.39.35c, 2017). Data reduction, scaling and absorption corrections were performed using CrysAlisPro (Rigaku, V1.171.40.76a, 2020). All non-hydrogen atoms were refined anisotropically. Hydrogen atom positions were either located from electron density maps and refined freely (or with restraints) using the non-spherical scattering factors or hydrogen atom positions were calculated geometrically and refined using the riding model.

Crystallographic data for compounds **1** and **2a** have been deposited with the Cambridge Crystallographic Data Centre, 12 Union Road, CB2 1EZ, United Kingdom (fax: +44–1223-336033; e-mail: deposit@ccdc.cam.ac.uk) and are available on request quoting the deposition number CCDC: 2046240 (compound **1**) and 2046241 (compound **2a**).

Crystallographic data of **1**. (C_30_H_48_O_6_). (H_2_O), *M*_*r*_ = 522.70, monoclinic, *P*2_1_ (No. 4), a = 7.54880(14) Å, b = 11.2160(2) Å, c = 16.9599(3) Å, *β* = 102.5498(18)^°^, *α* = *γ* = 90^°^, *V* = 1,401.64(5) Å^3^, *T* = 100.0(4) K, *Z* = 2, *Z'* = 1, *μ*(Mo K_*α*_) = 0.086 mm^−1^, 30,323 reflections measured, 12,736 unique (*R*_*int*_ = 0.0291) which were used in all calculations. The final *wR*_*2*_ was 0.1258 (all data) and *R*_*1*_ was 0.0460 (I > 2σ(I)).

Crystallographic data of **2a**. (C_30_H_48_O_6_). 2 (H_2_O), *M*_*r*_ = 540.72, monoclinic, *P*2_1_ (No. 4), a = 6.7500(3) Å, b = 12.2578(7) Å, c = 17.4707(12) Å, *β* = 98.740(6)^°^, *α* = *γ* = 90^°^, *V* = 1428.75(15) Å^3^, *T* = 110.1(8) K, *Z* = 2, *Z'* = 1, μ (CuK_*α*_) = 0.723 mm^−1^, 9,232 reflections measured, 3,582 unique (*R*_*int*_ = 0.0776) which were used in all calculations. The final *wR*_*2*_ was 0.1689 (all data) and *R*_*1*_ was 0.0648 (I > 2σ(I)).

**Bacterial Strains.** Strain details are compiled in Supplementary Table S1. Four strains were used for the *S. aureus agr* reporter panel: AH1677 (*agr* I), AH430 (*agr* II), AH1747 (*agr* III), and AH 1872 (*agr* IV). These reporter strains are respectively developed from the strains AH845, SA502A, MW2, and MN EV; each strain contains the plasmid pDB59, which encodes the *agr* yellow fluorescent protein reporter. Three *S. aureus* strains were used in the hemolysis assay: AH1263 (Erm sensitive USA300 LAC), AH1589 (the *hla::Tn551* mutant of AH1263), and AH1292 (the Δa*gr::tetM* mutant of AH1263). Three *S. aureus* strains were used in the δ-toxin quantification assay: NRS385, AH1263, and NRS249. Two *S. aureus* strains were used for biofilm studies: the clinical isolate UAMS-1 and its isogenic Δ*sarA* mutant UAMS-929, the biofilm production of which is severely retarded. These two strains are the only methicillin-sensitive *S. aureus* (MSSA) strains used; the rest are MRSA. All experiments were performed with treatments measured in µg/mL, and for castaneroxy A these values were then converted to µM. Therefore, the decimal places in the µM measurements of castaneroxy A are not meant to reflect the accuracy of the measurements.

**Minimum inhibitory concentration (MIC).** Clinical Laboratory Standards Institute (CLSI) M100-S23 guidelines for microtiter broth dilution testing were followed (M100, 2019). Briefly, overnight cultures in TSB were standardized by optical density (OD) to an OD_600_ of 0.0006, which corresponds to 5 × 10^5^ CFU/mL. Plate reading was done in a Cytation 3 multimode plate reader (Biotek). After treatment, plates were incubated at 37°C for 18 h under static conditions. Plates were read at an OD 600 nm at 0 and 18 h post-inoculation. All concentrations were tested in triplicate, and experiments were performed at least twice on different days to account for two biological replicates. The IC_50_ value of a treatment in a bacterial strain is defined as the lowest test concentration at which at least 50% growth inhibition was observed by culture optical density measurements. The MIC is defined as the lowest test concentration at which at least 90% growth inhibition is observed, equivalent to no visible growth in the wells.

**Reporter strains of inhibition of *agr* expression.** Fractions were tested for quorum sensing inhibitory activity against all four *agr* subtypes using previously described (Kirchdoerfer et al., 2011) *agr*::P3 YFP reporter strains AH1677 (type I), AH430 (type II), AH1747 (type III), and AH 1872 (type IV) (Figueroa et al., 2014). Overnight cultures in TSB and 10 µg/mL chloramphenicol were standardized to an OD_600_ of 0.0006 for working cultures. After treatment in 96-well black plates (Costar 3603), the plates were incubated at 37°C with shaking (1,000 rpm) in a humidified Stuart SI505 incubator (Bibby Scientific, Burlington, NJ). OD_600_ and fluorescence (top reading, 493 nm excitation, 535 nm emission, gain 60) readings were taken at 0 and 18 h post-inoculation.

**Growth curve.** An established drop plate method was used (Herigstad et al., 2001). Briefly, overnight cultures of *S. aureus* strain LAC (AH1263) in CAMHB were standardized to an OD_600_ of 0.0006, which corresponds to 5 × 10^5^ CFU/mL. Cultures were treated with either 2 or 10 µg/mL of castaneroxy A; a vehicle control of DMSO and a positive control of vancomycin (4 µg/mL) were used. Over the course of 24 h of growth in a 96-well plate in a humidified Stuart shaker at 37°C (1,000 rpm), culture dilutions were made at seven time points (0, 2, 4, 6, 8, 10, 24 h) in 1X phosphate-buffered saline (PBS) and 10 µL drops (in triplicate) were grown overnight on TSA to obtain colony-forming unit (CFU) counts. Optical density (OD_600_) measurements of the cultures were also taken at each time point.

**Hemolytic activity by red blood cell lysis assay.** The ability of castaneroxy A to inhibit quorum sensing in *S. aureus* was assessed by measuring the toxicity of treated culture supernatants to rabbit erythrocytes, as previously described (Quave et al., 2015). Briefly, overnight cultures of strain AH1263 (Erm sensitive USA300 LAC), AH1589 (the *hla::Tn551* mutant of AH1263), and AH1292 (the Δa*gr::tetM* mutant of AH1263) were inoculated 1:500 into TSB to make working cultures. Working cultures were transferred to a 96-well plate to receive treatment in quadruplicate. The three strains received one of four treatments—224C-F2, 224C-F2c, 224C-F2c-PF42, and **1**—at five concentrations: 2, 4, 8, 16, 32 µg/mL (equivalent to 3.97, 7.93, 15.86, 31.72, 63.45 µM for **1**). Vehicle control and untreated wells were included for all strains. The final well volume was 250 µL. After 8 h of incubation in a Stuart shaker (37°C, 1,000 rpm), well contents were transferred to 96-well filter plates, which were centrifuged stacked onto new plates at 2,000 *g* for 5 min. Supernatants (filtrates) were diluted 2-fold in triplicate seven times in new 96-well plates in 1.2X PBS with a final well volume of 30 µL.

Rabbit erythrocytes, obtained from defibrinated rabbit blood (Hemostat Laboratories, Dixon, CA) washed three times in 1.2X PBS, were resuspended in 1.2X PBS and were added 70 µL per well to the 96-well plates of supernatant dilutions. The plates were moved in circles by hand for mixing and then incubated statically at room temperature for 1.5 h. The plates were then read on a plate reader at OD_630_ for the detection of hemolysis. For each treatment-concentration in each strain, a 4-parameter logistic (4PL) fit was fit to the dilution vs OD data points using GraphPad Prism, and the midpoints of these curves were determined. The ratio of AH1263 vehicle 4PL midpoint to each treatment-concentration’s midpoint was determined and multiplied by 100.

**Quantification of δ-toxin by HPLC.** Inhibition of δ-toxin production in NRS385, a USA500 *agr* I HA-MRSA isolate, AH1263, a USA300 LAC isolate, and NRS249, an *agr* I isolate was tested, as reported previously (Quave and Horswill, 2018). In brief, 1.5 mL working cultures standardized to an OD_600_ of 0.0006 received sub-MIC_50_ concentrations of treatment. Supernatants were collected 15 h post-incubation at 37°C and shaking at 275 rpm; they were transferred to HPLC vials and stored at −20°C until HPLC testing. Samples were run on an Agilent 1260 Infinity system using a Resource PHE 1-mL (GE Healthcare, Uppsala, Sweden) analytical column, as previously described (Quave et al., 2015; Quave and Horswill, 2018). Two mobile phases were used: (A) 0.1% trifluoroacetic acid (TFA) in water, (B) 0.1% TFA in acetonitrile. The injection volume was 500 µL and the flow rate 2 mL/min. The 214 nm chromatograms were used for data processing. Two peaks corresponding to deformylated and formylated δ-toxin were integrated and their areas summed as a relative quantitative measure of δ-toxin presence.

**Biofilm assay.** A human plasma protein-coated assay was used, as previously described (Beenken et al., 2003; Quave et al., 2012). The strains used were UAMS-1, a PFGE USA200 osteomyelitis isolate (*agr* type III), and UAMS-929, the Δ*sarA* mutant of UAMS-1 that is deficient in producing biofilm; the latter strain was utilized as a positive control. Extract 220D-F2, shown to effectively inhibit biofilm formation in *S. aureus*, was used as an additional positive control (Quave et al., 2012). Treated working cultures in 96-well plates were statically incubated for 22 h at 37°C. Media was aspirated from the wells at the experimental endpoint. The biofilms were gently washed with 1X PBS, fixed with ethanol, stained with crystal violet, and rinsed with tap water to remove excess stain. The crystal violet stains were eluted into ethanol and transferred to a new 96-well plate. Stain quantification was achieved by an OD_595_ read on a Cytation 3 multimode plate reader (Biotek).

**Human keratinocyte toxicity.** Human immortalized keratinocytes (HaCaT cell line) were maintained as described previously (Quave et al., 2015). Upon reaching suitable confluency (90–95%), cells were standardized to 4 × 10^4^ cells/mL using a hemocytometer and 200 μL were seeded per well in a 96-well tissue culture-treated microtiter plate (Falcon 35–3,075). Plates were incubated for 48 h prior to media aspiration. Media was then added along with treatment. For the chemical cytotoxicity assay, treatment was an enriched extract or compound **1**. For the supernatant toxicity assay, treatment was spent bacterial supernatant (which was added as 20% of the well volume). After 24 h incubation, treatment-induced cytotoxicity was assessed by the CytoScan™ LDH Cytotoxicity Assay (G-Biosciences) following the manufacturer’s protocol.

**Animal Studies**. A previously described murine model of MRSA skin infection was used to determine the efficacy of **1** as an anti-infective (Muhs et al., 2017). All animal experiments described herein were approved by and conducted in accordance with the recommendations of the Animal Care and Use Committee at the University of Colorado Anschutz Medical Campus (IACUC protocol number 117217). One day prior to inoculation, the abdominal hair of 8-week-old female BALB/c mice was shaved and chemically removed with topical application of Nair for 1 min. LAC (USA300, AH1263) and its deletion mutant (LACΔ*agr*, AH1292) were grown in TSB media overnight at 37°C in a shaking incubator (200 rpm). Overnight LAC and Δ*agr* cultures were diluted 1:50 in fresh TSB media and allowed to grow to early log phase (∼2 h to an OD_600_ of 1.0). Cells were washed in sterile PBS and resuspended to achieve an inoculum of 1 × 10^8^ CFU. Groups were inoculated intradermally into the abdominal skin with 63 μL suspensions of either 1) 1 × 10^8^ CFU LAC and **1** (50 μg or 10 μg dissolved in DMSO), 2) LAC + DMSO vehicle control, or 3) 1 × 10^8^ CFU LACΔ*agr* + DMSO. Baseline body weights of the mice were taken prior to injection and each following day. For determination of lesion size, digital photos of skin lesions were taken with a Canon PowerShot ELPH180 camera and analyzed with ImageJ software for Mac. Inoculum CFU was verified by serial dilution, plating, and colony counting after overnight incubation of the plate. As no signs of distress were observed in the present study, all animals were euthanized via continuous administration of 100% CO_2_ at the experimental end point.

## Results

### Bioassay-Guided Isolation of Castaneroxy Compounds

The isolation of castaneroxy A (**1**) from the leaves of *C. sativa* followed a bioassay-guided fractionation scheme (Supplementary Figure S1). In brief, a double methanolic maceration of dried *C. sativa* ground leaves was performed, and the resulting filtered and dried crude extract was designated “224.” Crude extract 224 was sequentially partitioned against hexanes and ethyl acetate; the ethyl acetate partition, 224C, was fractionated as previously described via silica gel flash chromatography with three mobile phases: hexanes, ethyl acetate, and methanol. Flash fractions of 224C were produced, all given the prefix “F”. In order to determine which fraction was most likely to contain quorum sensing inhibitory compounds against *S. aureus*, a preliminary screen of the fractions was performed using a panel of four yellow fluorescent protein (YFP) *agr*::P3 reporter strains representing the four *agr* subtypes (Kirchdoerfer et al., 2011).

The fraction 224C-F2c exhibited the highest efficacy at quenching the reporter and was thus selected for further fractionation. Fraction 224C-F2c was fractionated by C-18 reverse phase high-performance liquid chromatography (HPLC) to produce 43 “preparative fractions,” all given the prefix “PF.” The 43 PFs were screened in the YFP *agr*::P3 reporter strain panel, which revealed that PF22-28 and 39-42 were the most bioactive (Supplementary Figure S2). Of these, fraction 224C-F2c-PF42 was selected for further fractionation via reverse phase HPLC, and this yielded 30 “subfractions,” all given the prefix “SF.” 224C-F2c-PF42-SF7 was compound **1**, and its structure and absolute configuration were determined by interpretation of nuclear magnetic resonance (NMR), mass spectrometry (MS), and single crystal X-ray diffraction (SCXRD) data. From 224C-F2c-PF42-SF6, a mixture of isomers of **1** (compounds **2a**, **2b**) was detected. Compound **2a** (major component) was crystallized and its structure was determined by X-ray diffraction analyses. Similar compounds to **1** and **2a**, **2b** have been reported previously (Cabrera et al., 1996; Banskota et al., 2000; Lacroix et al., 2009). Herein, the structure elucidation and MRSA antivirulence bioactivities of the castaneroxy compounds are described.

### Structure Elucidation of Castaneroxy Compounds

**FIGURE 1:**
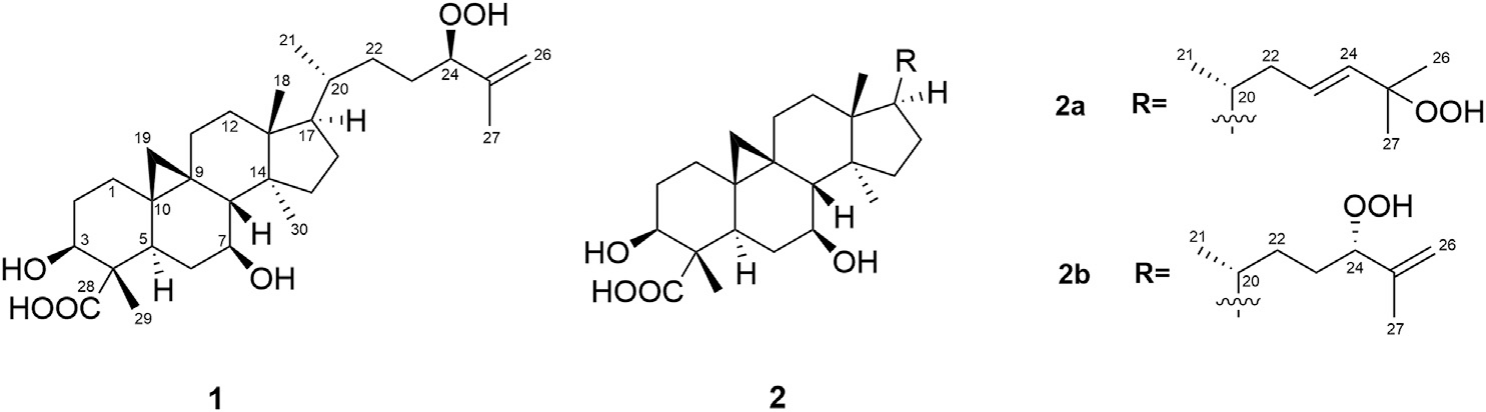
Structures of the isolated compounds (**1** and **2a**,**2b**).

Castaneroxy A (**1**) was obtained as a white amorphous solid (Figure 1) and the molecular formula was deduced to be C_30_H_48_O_6_ on the basis of HRESIMS data (*m/z* 503.3381, calcd for C_30_H_47_O_6_, [M–H]^-^) (Supplementary Figure S3), indicating seven degrees of unsaturation. Analysis of the ^13^C NMR data, DEPT-135, and HSQC spectra (Supplementary Figures S4–S6) revealed the presence of 30 carbons including three sp^2^ carbons (two olefinic carbons at δ_C_ 146.1 and δ_C_ 114.0 ppm and one carboxylic acid at δ_C_ 182.6 ppm) and 27 sp^3^ carbons, of which there were five methyls, ten methylenes, seven methines (three are oxygenated) and five quaternary carbons. The above satisfied two degrees of unsaturation, indicating the presence of a pentacyclic core.

The ^1^H NMR spectrum of **1** (Table 1; Supplementary Figure S7) displayed signals for five methyl groups at δ 1.71 (s), 1.10 (s), 1.00 (s), 0.95 (s), 0.90 (d, *J* = 6.4 Hz), one *exo*-methylene group at δ 4.91 (m) and 4.93 (dq, *J* = 1.6, 1.6 Hz), three oxymethine protons at δ 4.16 (dd, *J* = 6.8, 6.8 Hz), 4.01 (m) and 3.53 (brs), and two methylene protons with high-field resonances at δ 0.80 (d, *J* = 4.5 Hz) and 0.30 (d, *J* = 4.4 Hz), characteristic of a cyclopropyl group and in accordance with a cycloartane-type triterpenoid skeleton. The 1D NMR data of **1** were similar to those of the known compound musambin A (Lacroix et al., 2009), differing only in the position of one hydroxyl group, attached at C-7 (δ_H_ 3.53, brs; δ_C_ 70.7) in **1** instead of C-1 (δ_H_ 3.54, dd, *J* = 3.2, 2.8; δ_C_ 73.6) in musambin A.

**FIGURE 2:**
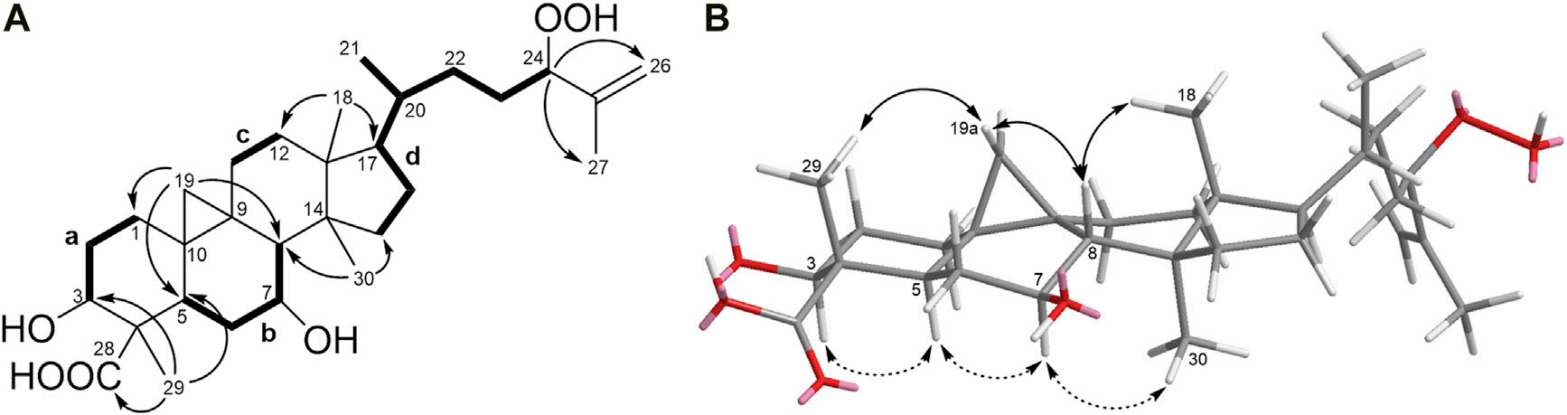
**(A)** ^1^H−^1^H COSY correlations and key HMBC correlations for compound **1**. **(B)** Key NOESY correlations of **1** (double-headed arrows). Solid arrows denote effects on the top face of the pentacyclic structure as shown; dashed arrows denote effects on the bottom face.

Detailed analysis of COSY and HMBC experiments (Supplementary Figures S8,S9) established the planar structure of **1** (Figure 1A). ^1^H-^1^H COSY spectral data revealed the presence of four spin systems (**a-d**): System **a** (–CH_2_–CH_2_–CH(OH)–), system **b** (–CH–CH_2_–CH(OH)–CH–), system **c** (–CH_2_–CH_2_–), and system **d** (–CH–CH_2_–CH–CH(CH_3_)–CH_2_–CH_2_–CH(OH)–) (Figure 2A). The HMBC correlations of H_3_-29 with C-3, C-5, and C-28 place H_3_-29 and a carboxylic acid at C-4 and connect systems **a** and **b**, while the correlations between H_2_-19 and C-1, C-5, and C-8 locate the cyclopropane ring at C-9 and C-10. Additionally, the HMBC cross peaks from H_3_-18 to C-12 and C-17 connect systems **c** and **d**, and the linkage of systems **d** and **b** was established on the basis of HMBC correlations from H_3_-30 to C-8 and C-15. Regarding the side chain, HMBC correlations observed from H-24 to C-26 and C-27 permit the lengthening of fragment **d** and confirm the presence of a hydroperoxide group at C-24, in agreement with the characteristic chemical shift value of this carbon at δ_C_ 90.9 ppm (Cabrera et al., 1996; Kato et al., 1996; Luo et al., 2005).

**FIGURE 3:**
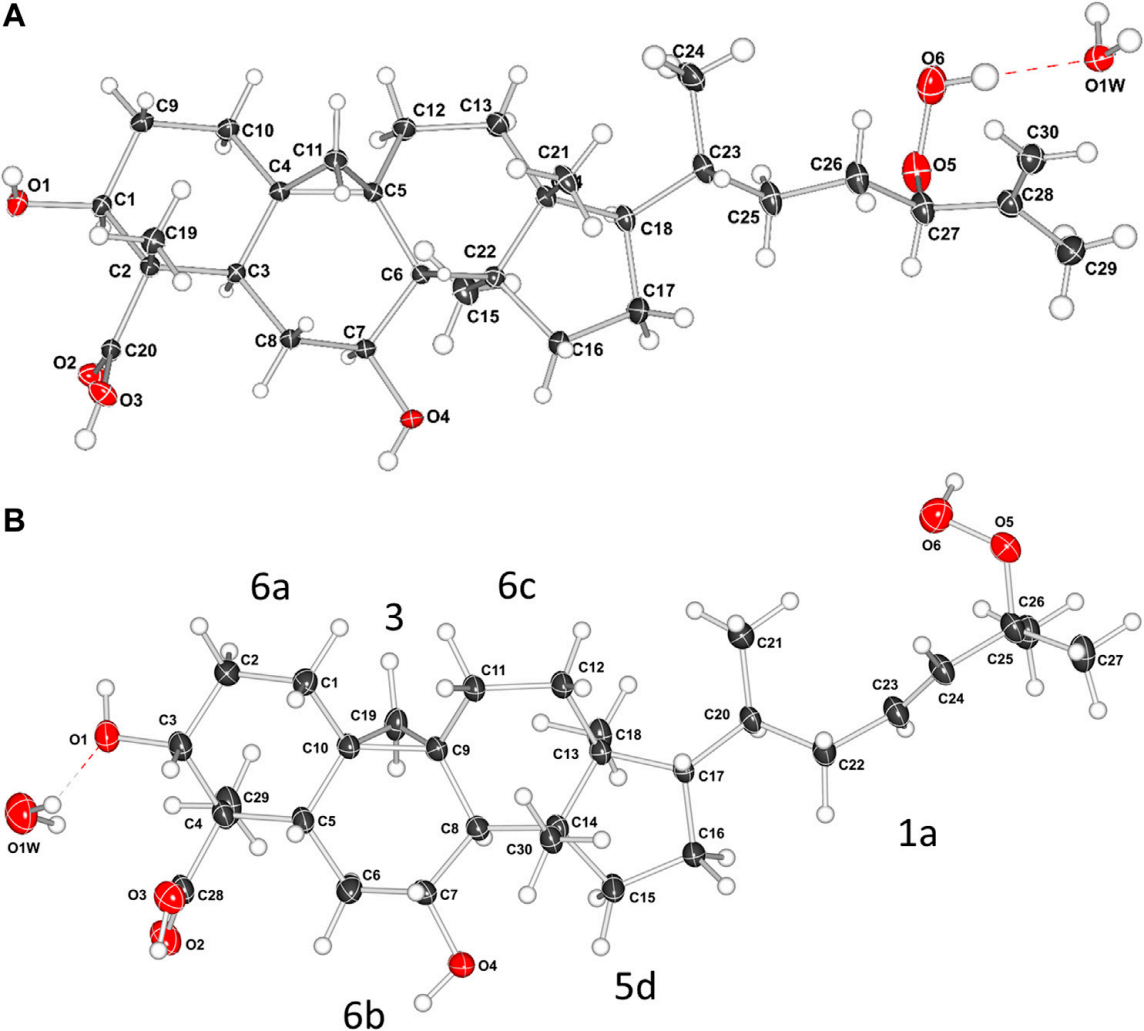
X-ray crystal structures of compound **1 (A)** and compound **2a (B)**. In **B**, the labels 6a, 3, 6b, 6c, 5d, and 1a refer to the rings and the alkyl chain; the numbers denote the ring size and the letters denote the position in the molecule.

The relative configuration of **1** (Figure 2B) was established based on NOESY experiments (Supplementary Figure S10). NOE correlations observed from H-5 to H-3/H-7, H-7 to H_3_-30 indicate that these protons are on the same face of the molecule, arbitrarily assigned to be alpha-oriented, whereas the NOEs between Ha-19 and H_3_-29/H-8 and between H-8 and H_3_-18 suggest that these protons are on the beta side of the molecule. To support the above deductions and determine the absolute configuration of **1**, an X-ray crystal structure was obtained (Figure 3A, Supplementary Information S2). The crystallographically determined torsional angles between the ^1^H−^1^H hydrogens corroborate the COSY correlations shown in Figure 2A. The absolute configuration of **1** was assigned as 3*S*, 4*S*, 5*R*, 7*S*, 8*S*, 9*S*, 10*R*, 13*R*, 14*S*, 17*R*, 20*R*, 24*R*, and this compound was named castaneroxy A (3β,7β-dihydroxy-24-hydroperoxy-cycloart-26-en-28-oic acid).

Compounds **2a** and **2b** were identified as an inseparable mixture of coeluting compounds (Figure 1). Exhaustive efforts to separate the mixture by several optimized conditions were unsuccessful. Thus, structure elucidation was performed on the mixture. HRESIMS data of **2a**/**2b** showed the same molecular formula as **1** (C_30_H_47_O_6_, [M - H]^-^, *m/z* 503.3392), suggesting the presence of isomers (Supplementary Figure S11). Comparison of ^1^H and ^13^C NMR for **2a**/**2b** (Table 1; Supplementary Figures S12–S14) with those for **1** showed that they shared the same cycloartane-type triterpenoid skeleton and suggested that the main differences are in the side chain. Detailed analysis of 1 and 2D NMR spectroscopic data allowed them to be identified and considered separately as two groups of signals, **2a** and **2b**.

The side chain structure of **2a** was determined based on its ^1^H-^1^H COSY and HMBC correlation data (Supplementary Figures S15,S16) and comparison of ^1^H and ^13^C chemical shifts with **1**. The most significant differences were the presence of olefinic signals (δ_H_ 5.57 and 5.62; δ_C_ 137.1 and 129.7 ppm) in **2a**, suggesting that a 1,2-disubstituted double bond replaced the *exo*-methylene moiety (δ_H-26_ 4.93 and 4.91, δ_C-25_ 146.1 and δ_C-26_ 114.0 ppm) in **1**, and suggesting the presence of a quaternary oxygenated carbon (δ_C-25_ 82.5 ppm) in **2a** instead of an oxymethine group (δ_H-24_ 4.16, 1H, dd, *J* = 6.8, 6.8 Hz; δ_c_ 90.9 ppm) in **1**. The position of the double bond was placed between C-23 and C-24 by the HMBC correlations from H-23 to C-25 and from H_3_-26/H_3_-27 to C-24. The downfield shift of C-25 confirmed that a hydroperoxy group was attached in this carbon (Sheu et al., 1996). Slow evaporation of a CH_3_OH solution of the mixture **2a**/**2b** yielded suitable crystals of **2a**, and a single-crystal X-ray diffraction study confirmed its structure and assigned the absolute configuration as 3*S*, 4*S*, 5*R*, 7*S*, 8*S*, 9*S*, 10*R*, 13*R*, 14*S*, 17*R*, 20*R* (Figure 3B; Supplementary Information S3). Compound **2a** was named castaneroxy B.

The NMR spectroscopic group of signals attributed to **2b** was almost identical to that of **1**, suggesting that they may be a pair of epimers. When comparing the ^1^H and ^13^C chemical shifts of both, the signals attributable to the side chains were found to be slightly different (Table 1), indicating that **2b** had to be the C-24 epimer of **1**. However, the structure and stereochemistry of **2b** could not be confirmed since the physical separation of mixture **2a**/**2b** was not achieved.

**FIGURE 4:**
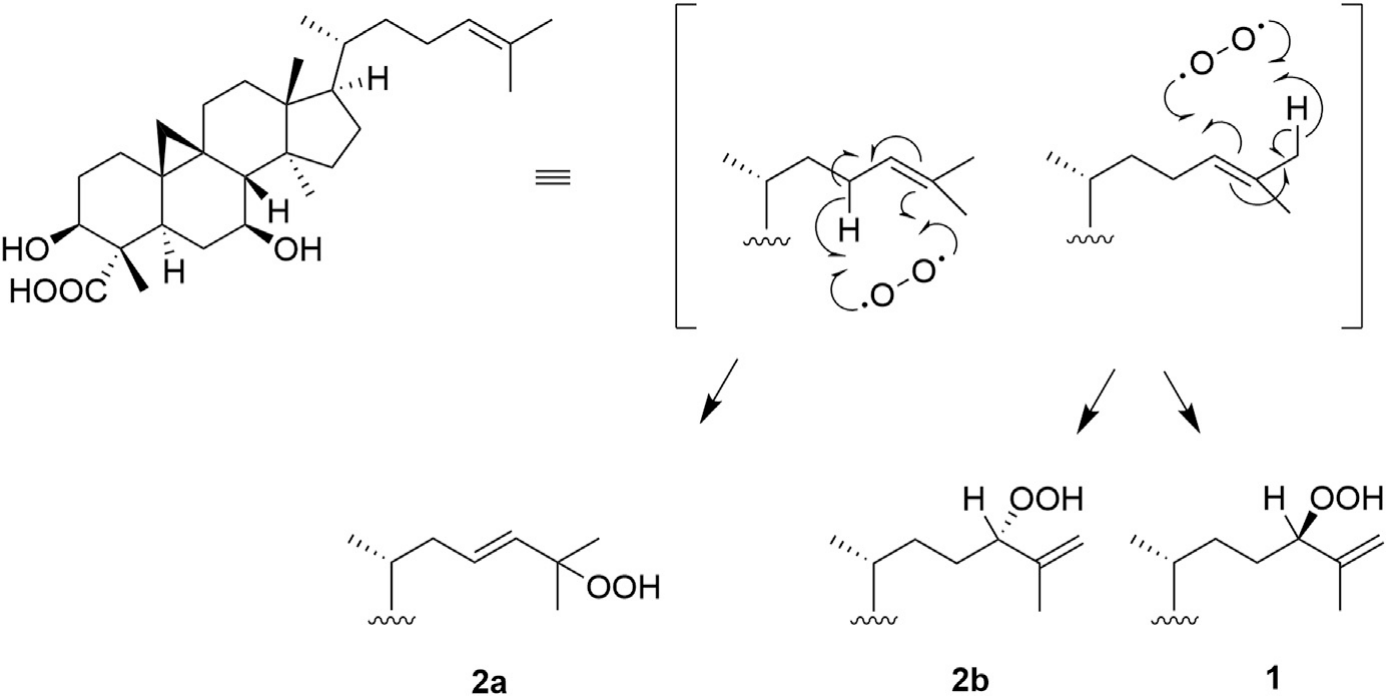
Proposed pathway for the formation of the castaneroxys from the hypothetical precursor. Based on the pathway proposed by Banskota et al. (Banskota et al., 2000).

The origin of compounds **1** and **2a**/**2b** can be understood by analyzing their biosynthetic pathway (Figure 4). Cabrera *et al.* proposed that these compounds might be generated via a naturally sensitized photooxygenation of olefinic precursors in the plant (Cabrera and Seldes, 1995). The reaction involves the formation of an allylic hydroperoxide from an olefin by a process involving abstraction of an allylic proton along with migration of the carbon-carbon double bond (Nickon and Bagli, 1961; Wasserman and Ives, 1981). Other studies have shown that in the presence of light, chlorophylls, due to their porphyrin sub-structures, are strong oxidation promotors and can act as photosensitizers to catalyze the photooxygenation of olefins to produce hydroperoxides (Kenney and Fisher, 1959; Matsushita and Terao, 1980; Yamauchi et al., 1982; Prein and Adam, 1996; Clennan, 2000). Previously, Δ^25(26)^-unsaturated hydroperoxy cycloartanes similar to **1** have been reported (Cabrera et al., 1996; Kato et al., 1996; Banskota et al., 2000; Lacroix et al., 2009), and in all such studies isomers thereof are concurrently reported. Additionally, Banskota *et al.* propose a possible pathway for the formation of hydroperoxy cycloartane isomers which occurs by the photooxygenation of a hypothetical precursor with a cycloartane-type skeleton (Banskota et al., 2000). As the reaction is non-stereoselective, product mixtures are commonly obtained.

The compound proposed as the hypothetical precursor of the castaneroxys has been previously reported (Popova et al., 2009). Its ^1^H spectrum exhibited a signal for one olefinic proton at δ_H_ 5.13 (t, *J* = 7.0 Hz, H-24) very similar to that observed in the ^1^H NMR data of the parent fraction 224C-F2c-PF42 (δ_H_ 5.09, t, *J* = 7.3 Hz), suggesting the presence of this precursor in the fraction studied and its possible participation in the biosynthetic pathway (Supplementary Figure S17).

**Castaneroxy A (1) acts on the *agr* system to inhibit virulence in MRSA**. To confirm the quorum sensing inhibitory activity of **1**, its concentration-dependent effects were examined on a number of *S. aureus* markers of virulence: *agr*::P3 activation, α-toxin and δ-toxin production, and culture supernatant toxicity to human cells. The IC_50_ of **1** on all strains to be examined was first determined in order to ensure subsequent testing at sub-IC_50_ concentrations (Table 2). The IC_50_ of **1** for the *S. aureus* strains UAMS-1, AH1263, AH1292, AH1589, and NRS835 is 64 µg/mL (126.90 µM). Therefore, the following assays were performed using **1** and its parent fractions at concentrations of up to 32 µg/mL (63.45 µM for **1**).

**TABLE 2:** IC_50_ and MIC values of castaneroxy A (**1**) against various *S. aureus* strains at 18 post-inoculation, based on optical density of growth cultures, reported both in µM and µg/mL for clarity.

Strain ID	IC	Castaneroxy A (µM)	Castaneroxy A (µg/mL)
UAMS-1	IC_50_	126.90	64
	MIC	126.90	64
AH1263	IC_50_	126.90	64
	MIC	253.80	128
AH1292	IC_50_	126.90	64
	MIC	253.80	128
AH1589	IC_50_	126.90	64
	MIC	253.80	128
NRS385	IC_50_	126.90	64
	MIC	253.80	128

**FIGURE 5:**
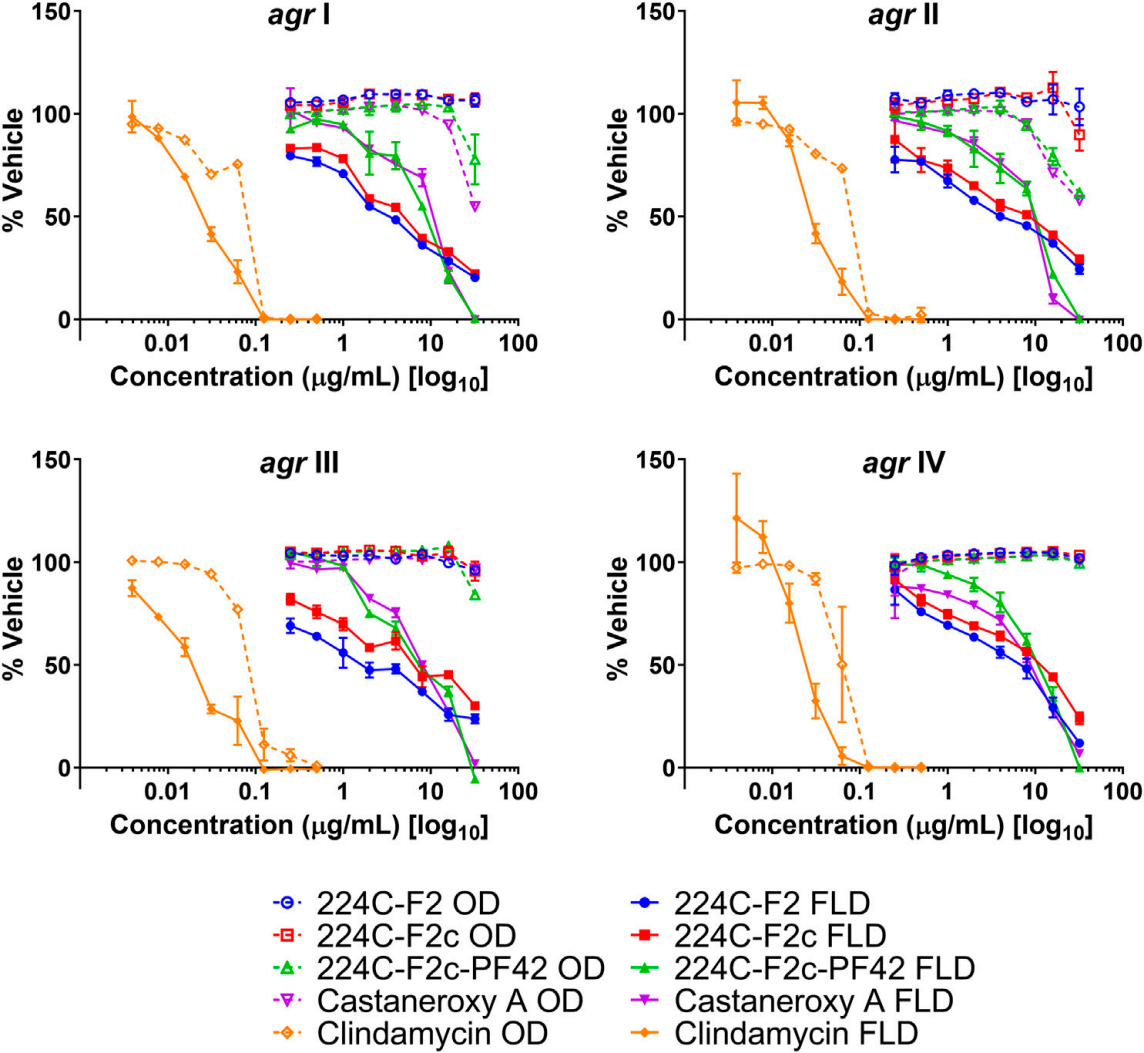
Castaneroxy A (**1**) and its parent fractions exert selective concentration-dependent inhibition of *agr*::P3 activation in all *agr* sub-types at 18 h post-inoculation. These were tested from 0.25–32 µg/mL on four *S. aureus agr*::P3 reporter strains: AH1677 (*agr* I), AH430 (*agr* II), AH1747 (*agr* III), AH 1872 (*agr* IV). All dashed lines represent optical density (OD) values, indicating growth; all solid lines represent fluorescence detection (FLD) values, indicating intensity of YFP detection as a result of *agr*::P3 activation. Clindamycin was used as a positive control for growth inhibition.

To confirm whether **1** targets the *agr* system in MRSA, it was assayed along with its parent fractions in the *agr*::P3 reporter strain panel, which was grown for 18 h (Figure 5). Compound **1** exhibited a concentration-dependent inhibition of YFP production in all strains in the panel, as did its parent fractions 224C-F2, 224C-F2c, and 224C-F2c-PF42 (referred to henceforth as F2, F2c, PF42). The results are summarized in Table 3. Across all *agr* types, the concentration response curves of F2 and F2c closely align with each other, suggesting that F2c contains most of the compounds that contribute to the effects of F2 in *agr*::P3 inhibition. The concentration response curves of PF42 and **1** are similarly closely aligned with each other in each reporter strain. One possible implication of this is that the castaneroxys in PF42 each exert similar activities on the *agr* system despite their differences in structure. Unlike F2 and F2c, PF42 and **1** exhibit growth delay activity at higher concentrations in *agr* Types I and II. This type of profile is typical of compounds that inhibit *S. aureus* regulatory pathways where a lower concentration of the compound exerts bioactivity but higher concentrations can be reached that cause growth delays. In the immediate case a growth delay is only observed in some, but not all, *S. aureus* strains. Collectively, these profiles in Figure 5 demonstrate that castaneroxy A inhibits *agr* expression independent of growth inhibitory effects.

**TABLE 3:** IC values of **1** and parent fractions on *agr* reporter strain fluorescence detection.

Strain ID	*agr* group	IC	Test agent (µg/mL)				
			224C-F2	224C-F2c	224C-F2c-PF42	1	1 (µM)
AH1677	I	IC_50_	4	8	16	16	31.72
		IC_90_	ND	ND	32	32	63.45
AH430	II	IC_50_	8	16	16	16	31.72
		IC_90_	ND	ND	32	32	63.45
AH1747	III	IC_50_	2	8	8	16	31.72
		IC_90_	ND	ND	32	32	63.45
AH1872	IV	IC_50_	8	16	16	16	31.72
		IC_90_	ND	ND	32	32	63.45

ND: IC not detected at the concentration range tested (0.25–32 µg/mL).

**FIGURE 6:**
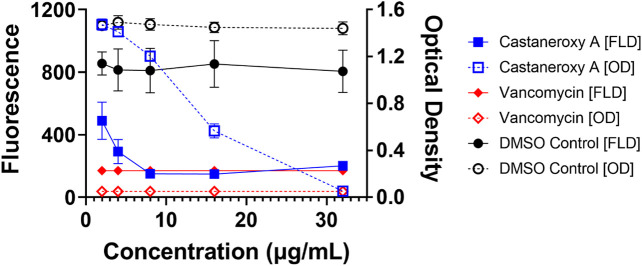
Higher concentrations of castaneroxy A inhibit reporter strain growth after exponential phase growth. The *agr* I reporter AH1677 was examined up to 8 h in a concentration-dependent test with vehicle (DMSO), positive control (vancomycin), and castaneroxy A. All dashed lines represent optical density (OD) values, indicating growth; all solid lines represent fluorescence detection (FLD) values, indicating intensity of YFP detection as a result of *agr*::P3 activation. Fluorescence and optical density measurements shown here are from 8 h post-inoculation in a Stuart shaker at 37°C at 1,000 rpm, with raw data values reported here. These data (*n* = 9) represent three independent experiments with three technical replicates.

**FIGURE 7:**
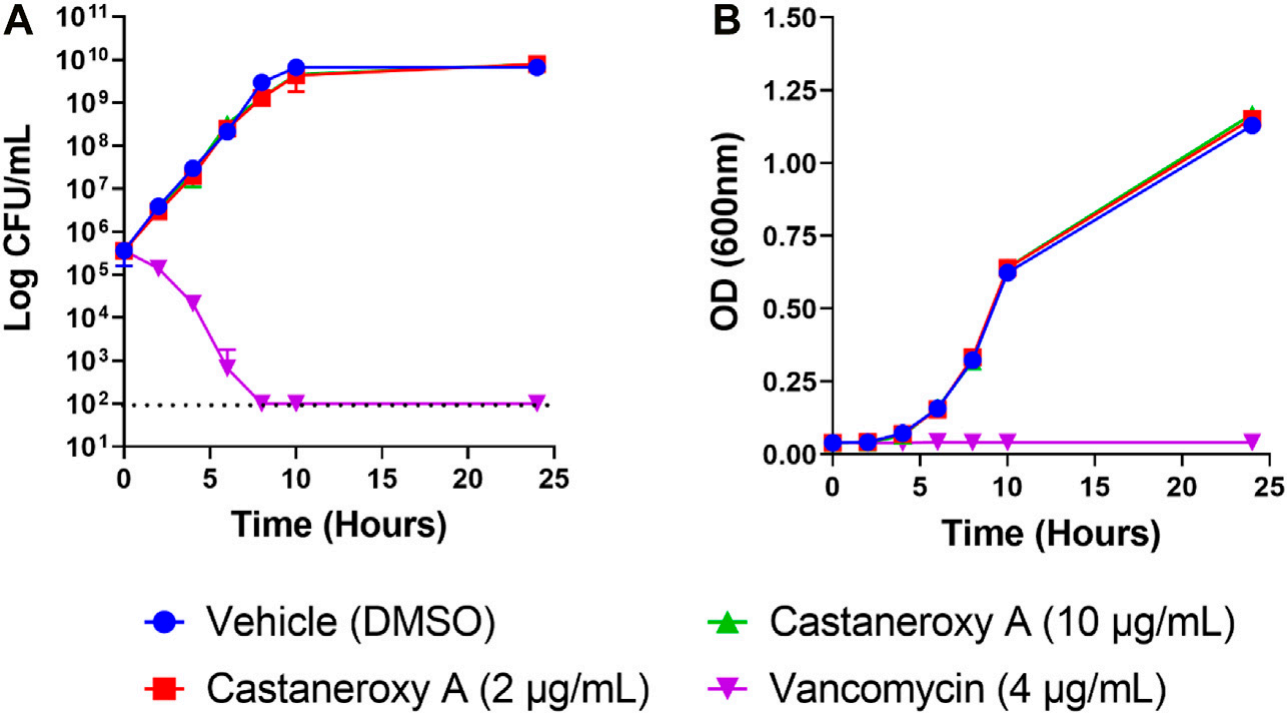
Effects of castaneroxy A (**1**) on *S. aureus* growth and survival. Growth curves were determined of *S. aureus* strain LAC treated with **1** (2 and 10 μg/mL), vehicle control (DMSO) and positive control (vancomycin, 4 μg/mL). Exponential-phase MRSA LAC (AH1263) was diluted in CAMHB by optical density to reach a final starting concentration of 5 × 10^5^ CFU/mL; this was confirmed by colony counts. **(A)** Viability was enumerated at the indicated time points by serial dilution plating. The limit of detection was 100 CFU/mL (indicated with dashed line), and no growth was detected for vancomycin for the time points of 8, 10 and 24 h. **(B)** Optical density (600 nm) of the cultures was also assessed at each time point. Experiments included three technical replicates and were repeated with new biological replicates, yielding the same results. Data presented here are from one experiment.

Sometimes, significant effects that compounds have on growth are seen during exponential growth and shortly thereafter, and they disappear after such a long time in stationary phase. Therefore the effects of **1** on the growth and fluorescence of the *agr* I reporter strain AH1677 up to 8 h post-inoculation were examined (Figure 6). During this period, growth effects are more pronounced, but they are largely limited to 16 and 32 µg/mL, while approximately 50% *agr*-inhibitory activity occurs at 2 µg/mL. Replicate samples were statistically analyzed for each 2-h interval (Supplementary Figures S18–S22). Additionally, a growth curve was determined examining LAC (AH1263) growth up to 24 h post-inoculation in the presence of **1** (Figure 7). The effects of 2 and 10 µg/mL of **1** were examined to determine growth inhibitory effects in the bioactive range for antivirulence effects. As confirmed by plate counts and optical density analyses, no growth inhibition occurred over the 24 h experiment, confirming that the *agr-*inhibitory activity observed in the bioactive range of 2–10 µg/mL is not due to any potential growth inhibitory effects of **1**.

**FIGURE 8:**
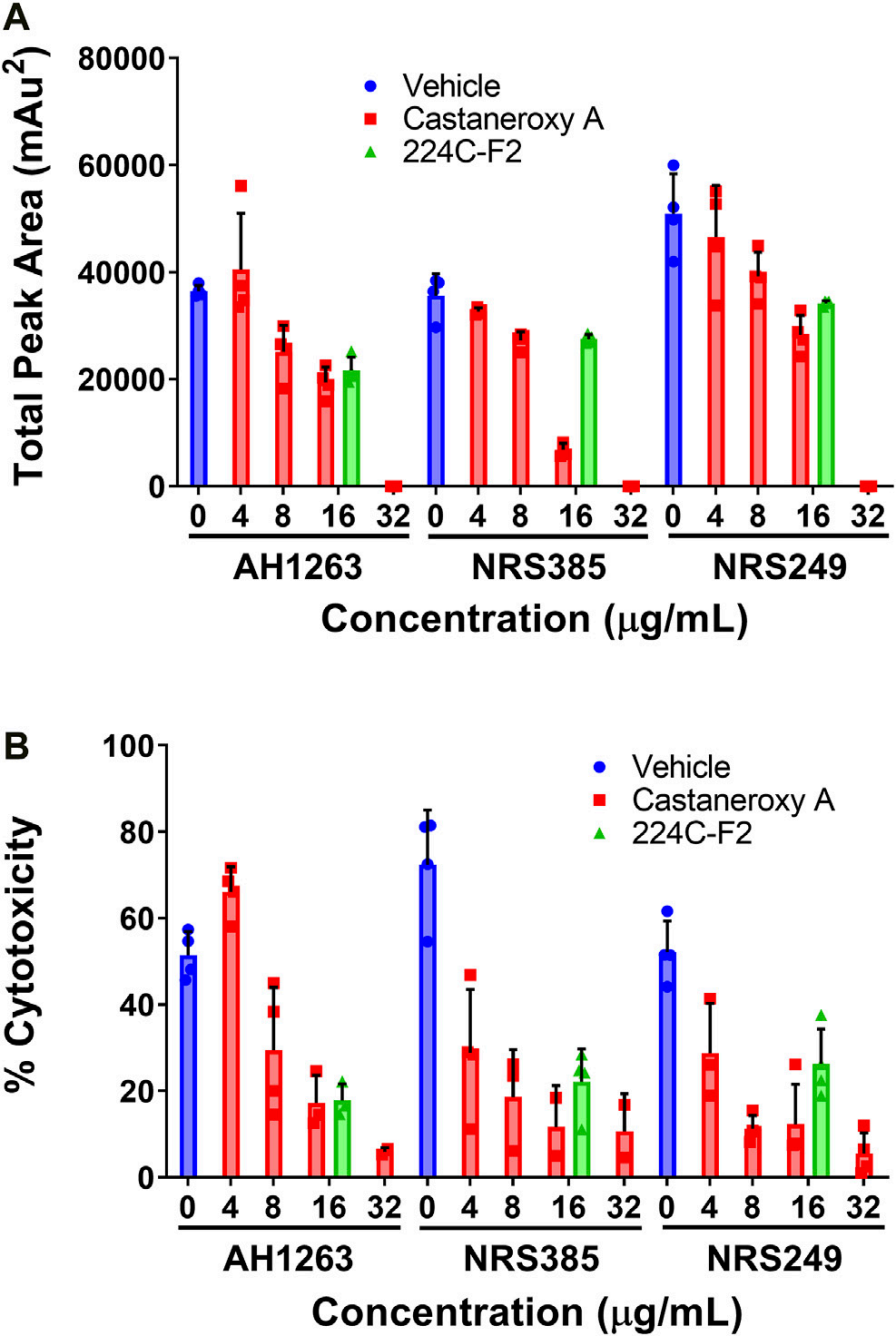
Castaneroxy A (**1**) inhibits δ-toxin production in a concentration-dependent manner. The strains AH1263 (LAC), NRS385, and NRS249, were cultured in the presence of increasing concentrations of **1**. **(A)** Supernatants from 15-hour cultures were analyzed by HPLC for the detection of δ-toxin. **(B)** HaCaT human keratinocytes were incubated in the presence of filtered culture supernatants (the same supernatants from part A) for 24 h; cytotoxicity was subsequently assessed by LDH assay. In both graphs, the dark-colored symbols represent the response of each replicate of a treatment-concentration. Each treatment-concentration was performed in quadruplicate; each experiment was performed in duplicate on separate days. Shown are the results of one of the duplicates of each experiment. Error bars represent SD.

With the ability of **1** established to inhibit transcriptional products of the *agr* system in MRSA independent of growth inhibitory effects, key translational products of *agr* were interrogated next. δ-toxin is a member of the phenol-soluble modulin (PSM) family of secreted peptides, and it is encoded by the *hld* gene (hemolysin delta), which is located on the RNAIII portion of the *agr* operon (Peschel and Otto, 2013; Otto, 2014b). Amphipathic and alpha-helical in structure, δ-toxin possesses a moderate capacity to lyse human neutrophils and contributes to synergistic hemolysis and PSM-mediated phenotypes such as bacteremia; a dedicated target of δ-toxin has yet to be determined (Peschel and Otto, 2013; Otto, 2014a). Effects of **1** on δ-toxin production were assessed in three high toxin producers: AH1263, an erythromycin (Erm) sensitive variant of USA300 strain LAC, NRS385, a USA500 *agr* I HA-MRSA isolate, and NRS249, an *agr* I isolate. In all three strains, culturing in the presence of increasing concentrations of **1** yields a concentration-dependent inhibition of δ-toxin production, as assessed by HPLC analysis of the culture supernatants (Figure 8A). At 32 µg/mL (63.45 µM), **1** exerts mild growth inhibitory activity and complete inhibition of δ-toxin production across the three strains. Since *hld* is located on RNAIII, inhibition of δ-toxin production represents a direct layer of evidence that **1** acts on the *agr* system, either directly or via an upstream regulator thereof.

In order to probe the inhibition of exotoxin production in general, immortalized human keratinocytes (HaCaT cells) were incubated in the presence of the same sterile-filtered supernatants as utilized above from AH1263, NRS385, and NRS249. Assessment of cytotoxicity by LDH assay reveals that in all strains, supernatants treated with increasing concentrations of castaneroxy A exert decreasing cytotoxicity on HaCaTs (Figure 8B). This observation indicates a concentration-dependent inhibition of exotoxin production by castaneroxy A. Different trends are observed between the three strains. In AH1263, the concentration-dependent decrease in HaCaT cytotoxicity mirrors very closely the concentration-dependent decrease in δ-toxin production. In both NRS385 and NRS249, however, the concentration-dependent effects between δ-toxin production and HaCaT cytotoxicity trends differ. At 4 µg/mL (7.93 µM), a concentration at which no significant decrease in δ-toxin production is affected, supernatant cytotoxicity to HaCaTs decreases significantly, indicating that inhibition of the production of toxins besides δ-toxin is achieved. The disproportional inhibition of HaCaT cytotoxicity as compared to δ-toxin production is also observable at 8 µg/mL (15.86 µM). Finally, in all strains, at 32 µg/mL (63.45 µM), a concentration at which δ-toxin production is completely shut down, some cytotoxic effect are still observed in HaCaTs, confirming that the production of other toxins persists.

**FIGURE 9:**
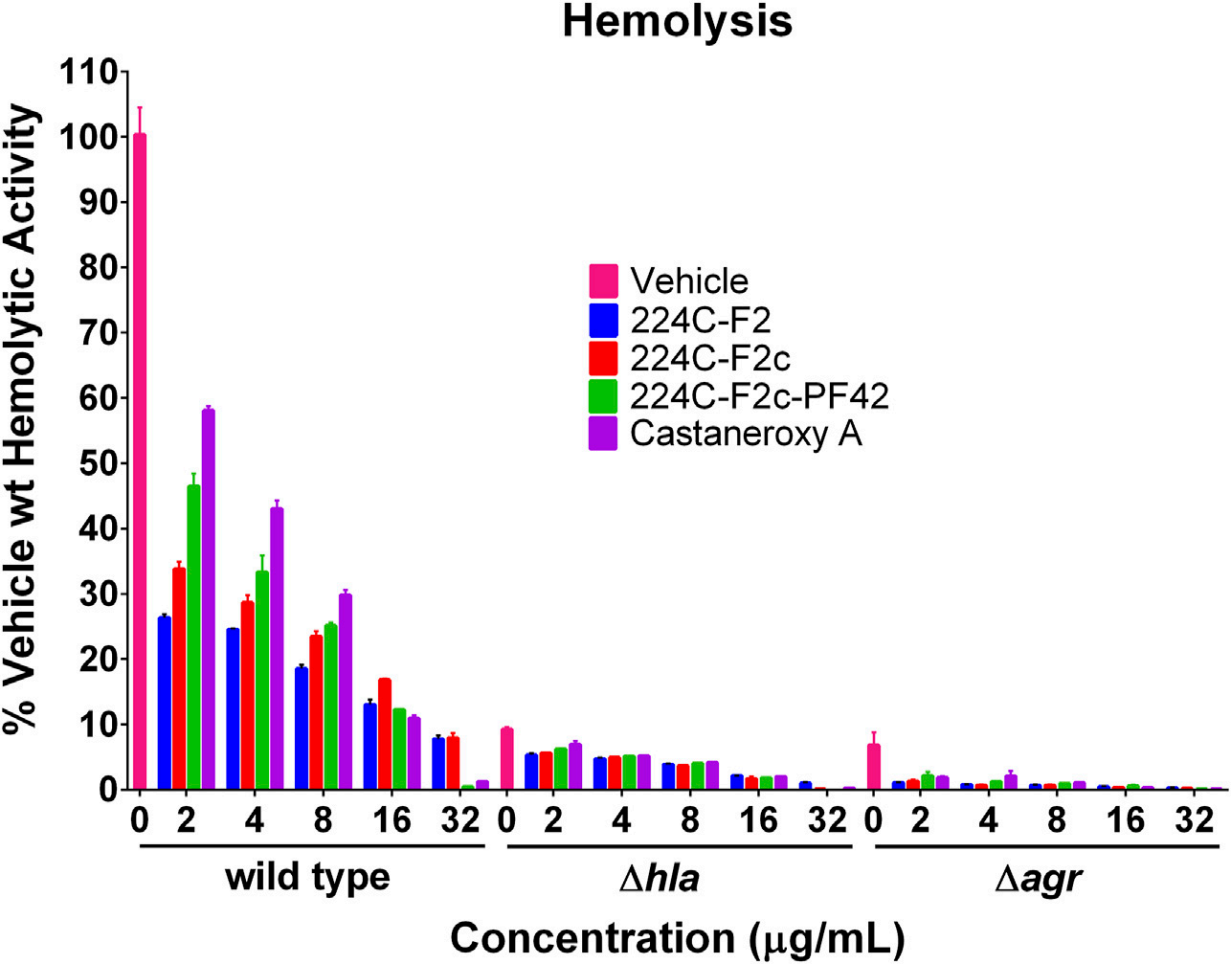
Castaneroxy A (**1**) and its parent fractions inhibit *S. aureus* hemolytic activity in wild-type, Δ*hla*, and Δ*agr* strains. The supernatants of treated cultures of three *S. aureus* strains were assessed for hemolytic activity against rabbit erythrocytes. Each treatment-concentration was performed in quadruplicate; the experiment was performed in duplicate on separate days. Shown is the result of one of the duplicates. Error bars represent SD.

Given the ability of **1** to inhibit the production of δ-toxin and other exotoxins, effects of **1** on α-toxin production were next assessed. Perhaps the best-studied *S. aureus* toxin, α-toxin has a mostly beta-sheet structure and is a member of the pore-forming beta-barrel toxin family (Otto, 2014b). It is encoded by the *hla* gene (hemolysin alpha) and possesses lytic activity for erythrocytes and a number of leukocytes, but not for neutrophils (Otto, 2014b). The expression of the *hla* gene is controlled by the *agr* system (Bronesky et al., 2016). To interrogate treatment effects on α-toxin production, we assessed the hemolytic activity of treated *S. aureus* culture supernatants on rabbit erythrocytes, which are extraordinarily sensitive to α-toxin (Dinges et al., 2000). Three strains were treated with increasing concentrations of **1**: AH1263 (LAC), here designated as “wild type,” AH1589, an *hla::Tn551* mutant of AH1263, and AH1292, an *Δagr::tetM* mutant of AH1263 (Figure 9).

In order to evaluate the role of chemical complexity in affecting hemolytic activity, the parent fractions of **1** were also tested. In the wild type strain, increasing concentrations of all treatments result in a concentration-dependent inhibition of hemolytic activity. A pattern is readily discernable, which also carries over to the other strains in a less pronounced fashion: increasing chemical complexity of the treatment results in increased inhibition of hemolytic activity. At 2, 4, and 8 µg/mL, 224C-F2 is more active than 224C-F2c, which is more active than 224C-F2c-PF42, which in turn is more active than **1** alone. This pattern breaks down at 16 and 32 µg/mL due to mild growth inhibitory effects of the latter two treatments, which increase apparent hemolytic activity. Increasing concentrations of treatment also cause a further inhibition of hemolytic activity of the Δ*hla* and Δ*agr* strains relative to vehicle treatment. Approximately 90% of hemolytic activity is negated by a deletion of either of these genes, confirming the extraordinary sensitivity of rabbit erythrocytes to α-toxin. That hemolytic activity is further inhibited in the Δ*hla* strain by treatment indicates that hemolytic toxins encoded by other genes, such as the PSMs, are still produced. Further inhibition in the Δ*agr* strain by treatment suggests that other systems besides *agr* might be targeted to some degree. The Sae system is one possible alternative target that could explain these phenotypes (Jenul and Horswill, 2018).

### Assessment of Other Effects of Castaneroxy A

**FIGURE 10:**
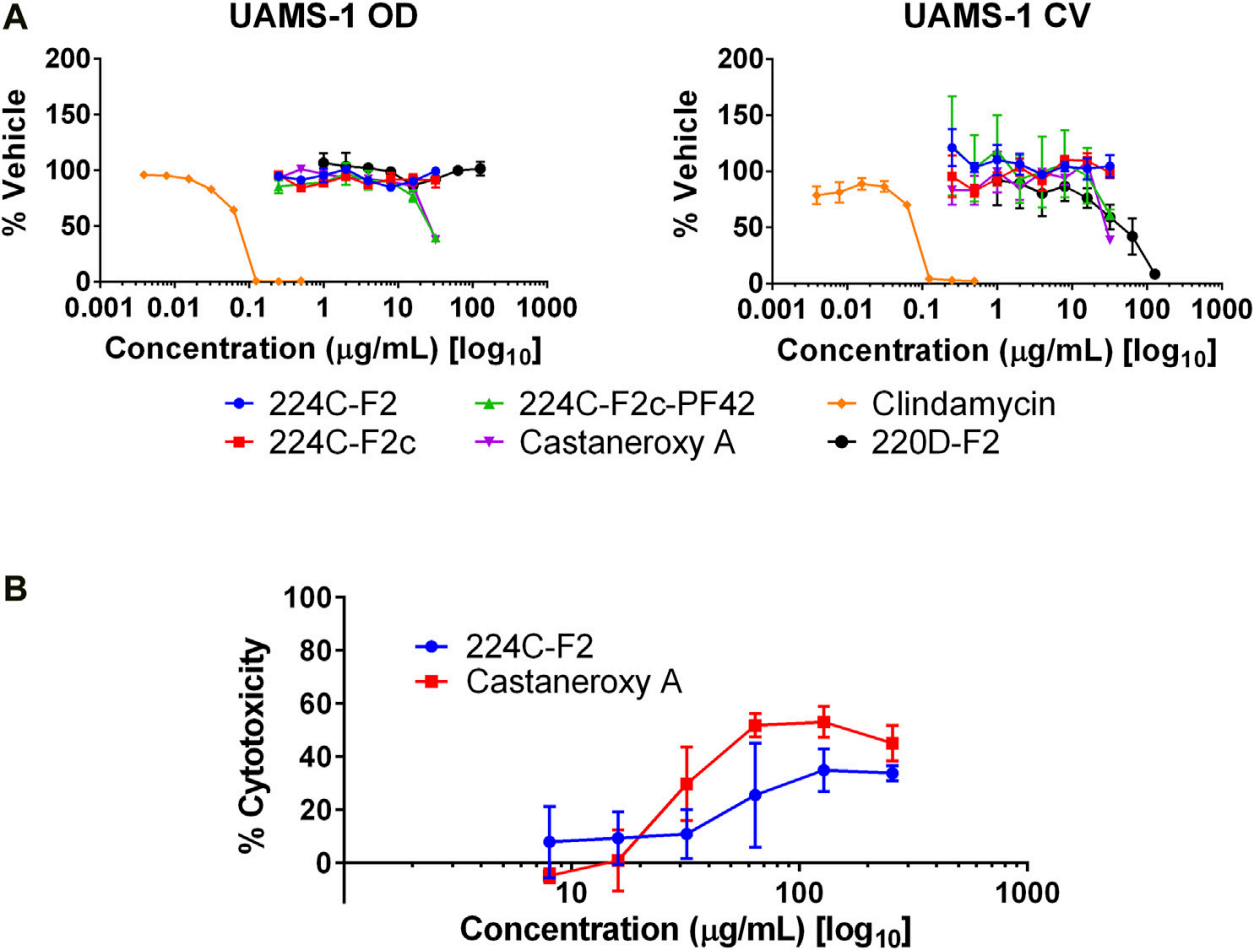
Other effects of castaneroxy A (**1**). **(A)** Compound **1** and its parent fractions do not inhibit biofilm formation in UAMS-1. Clindamycin was used as a control to demonstrate biofilm inhibition due to growth inhibition. The left graph shows optical density (OD), indicating growth as a result of treatment; the right graph shows crystal violet (CV) stain density retained by biofilms after treatment as a measure of biofilm production. **(B)** Concentration response curves demonstrate modest cytotoxicity of **1** and 224C-F2 against human keratinocytes (HaCaT cells).

With several lines of evidence presented for the inhibition of the *agr* system in MRSA, other effects were examined. The first was biofilm production. In staphylococci, the *agr* system controls the production of two groups of proteins involved in the structuring and dispersal of biofilms: PSMs and proteases (Dunman et al., 2001; Yao et al., 2005; Queck et al., 2008). In both *S. aureus* and *S. epidermidis*, *agr* mutants were shown to produce biofilms in an unstructured and extended fashion (Vuong et al., 2004; Periasamy et al., 2012). We sought to determine the concentration-dependent effect of **1** on biofilms of UAMS-1, a PFGE USA200 osteomyelitis *agr* III isolate, and its isogenic *sarA* mutant, UAMS-929, which is biofilm deficient. At concentrations ranging from 0.5–32 µg/mL, **1** and its parent fractions demonstrated no effect on biofilm production as assessed by crystal violet staining (Figure 10A).

Next, potential toxicity of **1** was investigated by incubation in cell culture with HaCaT human keratinocytes at concentrations from 8–256 µg/mL. After 24 h of incubation, **1** exerted cytotoxic activity greater than that of 224C-F2, as assessed by LDH assay (Figure 10B). Maximal cytotoxicity was achieved at 64 µg/mL (126.90 µM), a concentration at and beyond which it remained below 60%. At 32 µg/mL, or (63.45 µM), approximately 30% cytotoxicity is observed; no cytotoxicity is observed at lower concentrations. These data suggests that at the concentrations at which **1** inhibits the *agr* system in MRSA it exerts little to no cytotoxicity on mammalian cells. At higher concentrations, however, the cytotoxicity of **1** is evident and should be assessed in an expanded panel of safety studies in the future.

### Castaneroxy A Abates Dermonecrosis *In Vivo*

**FIGURE 11:**
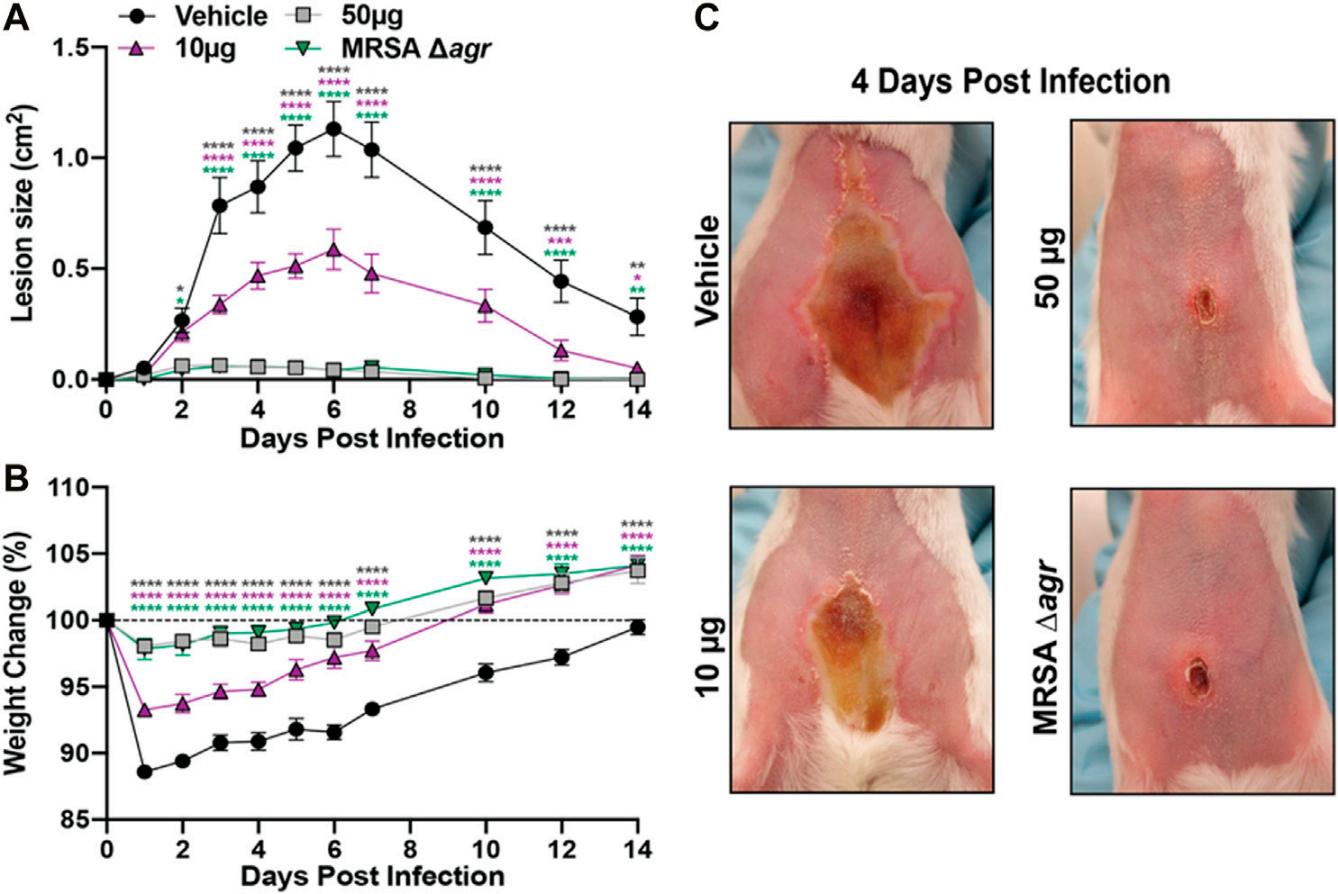
Castaneroxy A (**1**) protects murine skin from MRSA-mediated dermonecrotic injury and morbidity. **(A)** Dermonecrotic lesion size over time for mice challenged with MRSA and a 10 µg or 50 µg dose of **1** (or vehicle control) or MRSA Δ*agr* at the time of infection. **(B)** Weight change for indicated groups over the two-week infection period. **(C)** Representative images of dermonecrotic lesion size and severity at day 4 post-infection. Data are pooled from two independent experiments (*n* = 10 per group). Mean and SEM values are shown. Two-way ANOVA with Dunnett’s multiple comparison test was performed comparing the mean of the vehicle control to the mean of each test group for each day of measurements. A *p*-value <0.05 was considered significant. (*p ≤ 0.0332, **p ≤ 0.0021, ***p ≤ 0.0002, ****p ≤ 0.0001).

The antivirulence activity of **1** *in vitro* led us to assess its efficacy as an *in vivo* anti-infective. Using a previously established murine model of dermonecrosis (Muhs et al., 2017), BALB/c mice were intradermally challenged with MRSA (USA300) and a 50 µg or 10 µg dose of **1** or DMSO vehicle control at the time of infection. Compared to the vehicle control (DMSO), **1** reduced MRSA-mediated skin damage and animal morbidity in a dose-dependent manner (Figures 11A,B). Moreover, either dose of **1** was sufficient to significantly reduce dermonecrotic lesion size throughout the 2-week course of MRSA infection. Finally, a single 50 µg dose of **1** effectively reduced the dermonecrotic lesion area to nearly undetectable levels, similar to the MRSA *agr*-null mutant (Figure 11C). Taken together, these results demonstrate that a single dose of **1** can protect murine skin from MRSA infection-associated dermonecrotic injury and morbidity.

## Discussion

We have taken the ethnobotanical drug discovery approach from the discovery of a bioactive enriched extract of a plant used in traditional medicine in the Mediterranean and Balkans to the isolation of a bioactive cycloartane-type triterpenoid, castaneroxy A (**1**) (Cox and Balick, 1994). Cycloartane triterpenoids have previously been reported with a range of activities including moderate to high antibacterial and anti-cancer activity (Dinda et al., 2010). Compound **1** is a novel compound reported here for the first time and represents the first cycloartane triterpenoid reported to possess antivirulence activity against MRSA. Until now, hydroperoxy cycloartanes have mostly been reported with antimicrobial and anti-cancer activity (Vil et al., 2019).

The crystallographic data allowed for a detailed conformational analysis of all the ring systems in **1** and **2a**. The molecular structure of **1** contains a scaffold consisting of a polycyclic ring system with five fused rings. The compound has a chiral (3*R*)-2-methylhept-1-ene-3-peroxol group which is attached to the polycyclic ring system at the cyclopentane ring. The presence of an alkene group with a reactive peroxide group may appear to be unusual in a natural source but more than 1,000 peroxides from nature have been isolated and structurally characterized (Dembitsky, 2015). There are 12 chiral centers in the molecule. Following the notation in Figure 3B, the cyclopropane-ring (3) shares an edge with a cyclohexane-ring (6b) which in turn shares edges with two rings 6a and 6c (forming four loops). The ring 6c shares an edge with the cyclopentane-ring (5d) and a corner of the cyclopropane-ring (3), also a four-looped circuit. The ring 5d connects to the alkyl chain (1a).

The ring 5d is puckered and is best described as having a twisted (T) conformation on bond C13-C14. This is very similar to the perfect sin-form (Evans and Boeyens, 1989). The substituents C12, C18, C8, C30, C20 are Eq, Ax, Eq, Ax, and Eq respectively (Eq meaning equatorial and Ax meaning axial). Ring 6a is very close to an ideal chair conformation (C). The substituents O1, C28, C29, C6, and C9 are Eq, Eq, Ax, Eq, Eq respectively. The cyclohexane-ring (6b) is more planar because of the bridging cyclopropane ring and the extent of puckering is less. It has 25% contributions each from the ideal boat and twisted forms and a 50% contribution from the B_2g_ (chair) conformation. The cyclohexane-ring (6c) is strongly puckered with a 53% contribution from the sin(E_2u_) (twisted) conformation and 24% contributions each from the ideal boat and a chair forms. The peroxy group donates a strong hydrogen bond to a water molecule.

The molecular structure of **2a** contains the same polycyclic ring system with five fused rings. However, the compound has a chiral (3*E*)-2-methylhept-3-ene-2-peroxol group at the cyclopentane ring 5d. There are 11 chiral centers in the molecule. The conformations of the polycyclic ring system for both compounds are virtually the identical and will be discussed for 1a only. The hydrogen bonding with the peroxy group is different to 1a and a water molecule is not positioned to accept a strong hydrogen bond from the peroxide. The Flack parameter could not be used to assign the chirality for this X-ray data (although it could be tentatively assigned from the X-ray data using the Bijvoet pairs and from Bayesian statistics). On the basis of the similarity of the two structures and the identical sources of the compounds the chirality was determined to be the same for both compounds.

In comparison to its parent fraction 224C-F2, **1** demonstrates similar activities across a range of assays assessing inhibition of MRSA *agr* transcriptional and translational products *in vitro*. While **1** shows slightly lower potency in the inhibition of *agr*::P3 activation and hemolytic activity, it shows similar potency in the inhibition of δ-toxin production and supernatant toxicity in MRSA. Given that the *hld* gene is on the RNAIII portion of the *agr* operon, which is transcribed upon activation of the P3 promotor on *agr*, it is expected that concentration-dependent effects would match between inhibition of *agr*::P3 activation and δ-toxin production. Indeed, at the IC_50_ (31.72 µM; 16 µg/mL) and IC_90_ (63.45 µM; 32 µg/mL) of **1** for *agr*::P3 inhibition in *agr* I, **1** inhibits *agr* I δ-toxin production by about half and by 100%, respectively, across the three strains assayed.

224C-F2 exerted biofilm inhibitory activity at concentrations higher than those at which **1** demonstrates antivirulence activity (Quave et al., 2015). In turn, **1** does not exert any biofilm inhibitory activity at concentrations below growth inhibitory effects. Some other antivirulence compounds have, however, demonstrated concurrent antivirulence and anti-biofilm effects. Studies on MRSA antivirulence compounds, such as that examining alizarin and related compounds, even show changes in gene expression related to biofilm production as well as toxin production as a result of treatment (Lee et al., 2016). Compared to such compounds, **1** exhibits specific antivirulence activity.

Another notable trend is that observed in the inhibition of hemolytic activity by **1** and its parent fractions. In the case of this bioassay, decreasing chemical complexity led to decreased activity over a concentration range. While **1** exhibits similar potency to 224C-F2 in the inhibition of δ-toxin production and supernatant toxicity, a difference in potencies is pronounced in the *agr*::P3 activation screen which guided fractionation. While in some cases fractionation enriches the abundance of bioactive compounds, fractionation may also cause decreases in bioactivity, particularly in chemical compositions in which synergy between compounds is responsible for bioactivity (Caesar and Cech, 2019; Dettweiler et al., 2020). In the immediate case, what is seen is a decrease in activity against some markers of MRSA virulence but not others due to fractionation. This points to a number of possibilities, including the potential of the more chemically complex fractions to contain chemistries that inhibit other virulence regulators, such as SarA, which regulates the *agr* system by activating *agr* P2 expression, or SaeRS, which like *agr* also regulates α-toxin production (Jenul and Horswill, 2018).

Castaneroxy A demonstrates comparable bioactivity to recently-discovered *S. aureus* quorum sensing inhibitor leads that have been tested in mice (Sully et al., 2014; Daly et al., 2015; Todd et al., 2017; Tan et al., 2018; Parlet et al., 2019; Tang et al., 2020), as summarized in Supplementary Table S2. Compared to the natural products apicidin and OHM, **1** generally demonstrated higher potency in the *agr*::P3 reporter panel (Daly et al., 2015; Parlet et al., 2019). Compound **1** also demonstrated higher toxicity than apicidin, OHM, and ambuic acid to the reporter panel and LAC (Daly et al., 2015; Todd et al., 2017; Parlet et al., 2019). Like the castaneroxys, another set of *agr* system inhibitor leads were identified in an enriched plant extract: the triterpenoid acids 3-oxo-olean-12-en-28-oic acid, 3-oxotirucalla-7,24*Z*-dien-26-oic acid and 3α-hydroxytirucalla-7,24*Z*-dien-27-oic acid (Tang et al., 2020). Compared to **1**, these triterpenoid acids generally exhibit lower toxicities against a panel of MRSA strains, higher potencies against the *agr*::P3 reporter panel, lower potencies at δ-toxin inhibition, and lower or equal efficacies in a mouse model of MRSA skin infection.

Savirin is another quorum sensing inhibitor that exerts similar activity to that of **1** both *in vitro* and *in vivo* (Sully et al., 2014). A unique set of experiments performed in the savirin study are those assessing *S. aureus* membrane integrity and membrane potential upon treatment. These are properties known to be impacted by antibiotics and thus lead to decreased *agr* expression (Attia et al., 2010; Dengler et al., 2011). In fact, a recent study reports that the membrane-active lipopeptide, C_10_OOc_12_O, at sub-growth inhibitory concentrations to MRSA causes mild membrane depolarization with an accompanying reduction of lipase and α-toxin outputs and resensitization to oxacillin (Hershkovits et al., 2019). Another study on oxacillin showed that sub-growth inhibitory concentrations of the β-lactam antibiotic caused an altered cell membrane architecture in MRSA while also downregulating *agr*, resulting in reduced virulence *in vitro* and *in vivo* and enhanced killing *in vivo* (Waters et al., 2016). While the mechanism by which **1** inhibits MRSA quorum sensing has not been probed in this study, its relatively low IC_50_ values across assays measuring virulence inhibition justify future inspection of cell membrane effects. Other possible targets not connected directly to the *agr* system are two-component systems and other regulators that impact *agr* P2 and P3 activation (Jenul and Horswill, 2018).

Further development of **1** as a MRSA quorum sensing inhibitor would entail elucidating mechanism of action and chemical modification to improve pharmacodynamic and pharmacokinetic properties. Indeed, given the moderate growth delay effects on some MRSA strains at higher treatment concentrations, the range of concentrations at which **1** functions specifically as a quorum sensing inhibitor can be improved. A much wider range of antivirulence activity was observed in the enriched extract 224C-F2. While medicinal chemistry could improve this as well as pharmacokinetic parameters, the process for modifying a compound as stereochemically complex as **1** is particularly challenging (Morrison and Hergenrother, 2014; Rodrigues et al., 2016). Mechanism of action studies and preliminary pharmacokinetic studies to assess bioavailability and metabolic stability would inform as to the potential of **1** for lead optimization as a clinical drug.

The proposed precursor of **1** has been reported to be first isolated from Cretan propolis with an MIC against *S. aureus* ATCC 25923 of 700 µg/mL. This precursor may also contribute to the ethnobotanical anti-infective value of *C. sativa* leaves in Italian traditional medicine. In screening the PFs of 224C-F2c against the *agr*::P3 reporters (Supplementary Figure S2), another set of PFs (PF22-28) also proved active and may contain further compounds that contribute to—perhaps by synergy—to the anti-infective value of the plant. Indeed, this synergy of active compounds in 224C-F2 may explain its ability to inhibit MRSA virulence at concentrations significantly below growth inhibitory concentrations. Therefore, a logical next step in developing **1** as an antivirulence compound should involve exploring this potential synergy.

The *C. sativa* leaves from which the castaneroxys were identified were collected in Italy. *Castanea* species are also found in the United States, and the question of whether these same hydroperoxy cycloartane triterpenoids or their precursors are produced in them and contribute to their ethnobotanical anti-infective value merits investigation. Native American peoples of North America have historically utilized the leaves of various *Castanea* species in traditional medicinal preparations for a host of indications, infection-related and otherwise. For example, the Cherokee have used the leaves of *C. dentata* in preparations for treating coughs, sores, and heart troubles (Hamel and Chiltoskey, 1975); they have also used the leaves of *C. pumila* to treat headaches, fevers, chills, and cold sweats (Taylor, 1940; Hamel and Chiltoskey, 1975). The Mohegan people have used the leaves of *C. dentata* in preparations for treating rheumatism, colds, and whooping cough (Tantaquidgeon, 1928; Tantaquidgeon, 1972).

We have demonstrated the isolation of **1** from the leaves of *C. sativa* as well as its confirmation as an inhibitor of the *S. aureus agr* system. Our findings both identify **1** as a potential lead for MRSA antivirulence drug development and confirm the ethnobotanical anti-infective value of *C. sativa* as used in Italian traditional medicine. We have followed the classical framework of ethnobotanical drug discovery (Cox and Balick, 1994). Under this framework, traditional ethnobotanical knowledge focuses plant extract screening to extracts of plants with proven bioactivities of interest, and subsequent bioassay-guided fractionation of extract hits leads to the isolation of bioactive constituent compounds. Following this framework, we were able to tap into underexplored natural chemistries used by people for generations for the treatment of infections. Using state-of-the-art bioassays such as the *agr*::P3 reporter strain panel, those natural chemistries were screened for the innovative antivirulence bioactivity against MRSA as an anti-infective alternative to classical growth inhibition, which inevitably leads to antibiotic resistance. Whether **1** and other products of such screenings can successfully improve therapeutic outcomes in the treatment of MRSA infections—either alone or as an adjuvant to antibiotics—remains to be determined.

## Data Availability

The datasets presented in this study can be found in online repositories. The names of the repository/repositories and accession number(s) can be found in the article/Supplementary Material.
