# Supplementary material for: Castaneroxy A From the Leaves of Castanea sativa Inhibits Virulence in Staphylococcus aureus
Source: Front Pharmacol. 2021 Jun 28;12:640179. doi: 10.3389/fphar.2021.640179 (PMC8274328; doi:10.3389/fphar.2021.640179)
Supplement: Supplementary file 2 [file DataSheet3.DOCX]

Supplementary Material

# Supplementary Data

**Castaneroxy A from the leaves of *Castanea sativa* inhibits virulence in *Staphylococcus aureus***

Akram M. Salam^1^, Gina Porras^2^, Young-Saeng K. Cho^3^, Morgan M. Brown^3^, Caitlin J. Risener^1^, Lewis Marquez^1^, James T. Lyles^2^, John Bacsa^4^, Alexander R. Horswill^3^, Cassandra L. Quave^2,5,6,*^

^1^Program in Molecular and Systems Pharmacology, Laney Graduate School, Emory University, Atlanta, GA 30322.

^2^Center for the Study of Human Health, Emory University, Atlanta, GA 30322.

^3^Department of Immunology and Microbiology, University of Colorado Anschutz Medical Campus, Aurora, CO 80045.

^4^Department of Chemistry, Emory University, Atlanta, GA 30322.

^5^Department of Dermatology, Emory University School of Medicine, Atlanta, GA 30322.

^6^Antibiotic Resistance Center, Emory University, Atlanta, GA 30322.

^*^To whom correspondence may be addressed. Email: cquave@emory.edu

INDEX

Figure S1. Fractionation scheme of methanolic extract of *C. sativa* leaves. 3

Figure S2. Reporter strain screen of *agr*::P3 expression indicates fraction 224C-F2c and 224C-F2c-PF42 are potential sources of quorum sensing inhibitors*.* 4

Figure S3. ESI-MS negative mode spectrum and empirical formula calculations of compound 1. 5

Figure S4. ^13^C NMR spectrum of 1 in CD_3_OD. 6

Figure S5. DEPT-135 spectrum of 1 in CD_3_OD. 7

Figure S6. HSQC spectrum of 1 in CD_3_OD. 8

Figure S7. ^1^H NMR spectrum of 1 in CD_3_OD. 9

Figure S8. COSY spectrum of 1 in CD_3_OD. 10

Figure S9. HMBC spectrum of 1 in CD_3_OD. 11

Figure S10. NOESY spectrum of 1 in CD_3_OD. 12

Figure S11. ESI-MS negative mode spectrum and empirical formula calculations of compounds 2a, 2b. 13

Figure S12. ^1^H NMR spectrum of compounds 2a, 2b in CD_3_OD. 14

Figure S13. ^13^C NMR spectrum of compounds 2a, 2b in CD_3_OD. 15

Figure S14. HSQC spectrum of compounds 2a, 2b in CD_3_OD. 16

Figure S15. COSY spectrum of compounds 2a, 2b in CD_3_OD. 17

Figure S16. HMBC spectrum of compounds 2a, 2b in CD_3_OD. 18

Figure S17. Zoomed in ^1^H spectrum of 224C-F2c-PF42. 19

Figure S18. Effects of 2 μg/mL castaneroxy A and parent fractions on AH1677 over 8 h. 20

Figure S19. Effects of 4 μg/mL castaneroxy A and parent fractions on AH1677 over 8 h. 21

Figure S20. Effects of 8 μg/mL castaneroxy A and parent fractions on AH1677 over 8 h. 22

Figure S21. Effects of 16 μg/mL castaneroxy A and parent fractions on AH1677 over 8 h. 23

Figure S22. Effects of 32 μg/mL castaneroxy A and parent fractions on AH1677 over 8 h. 24

Table S1. Profiles of *S. aureus* strains used in this study. 25

Table S2. Comparison of select activities of other *S. aureus* quorum sensing inhibitors. 26

# Supplementary Figures and Tables

## Supplementary Figures

**Figure S1.** Fractionation scheme of methanolic extract of *C. sativa* leaves. The fractionation of the crude methanolic extract of *C. sativa* leaves, 224, was done in four rounds using different methods: liquid-liquid partitioning was performed first, then normal phase flash chromatography, and then two iterations of reverse phase HPLC. Percent yield values are indicated as well as percent vehicle values for *agr*::P3 activation in 16 µg/mL treatment in AH1677 (*agr* Type I).


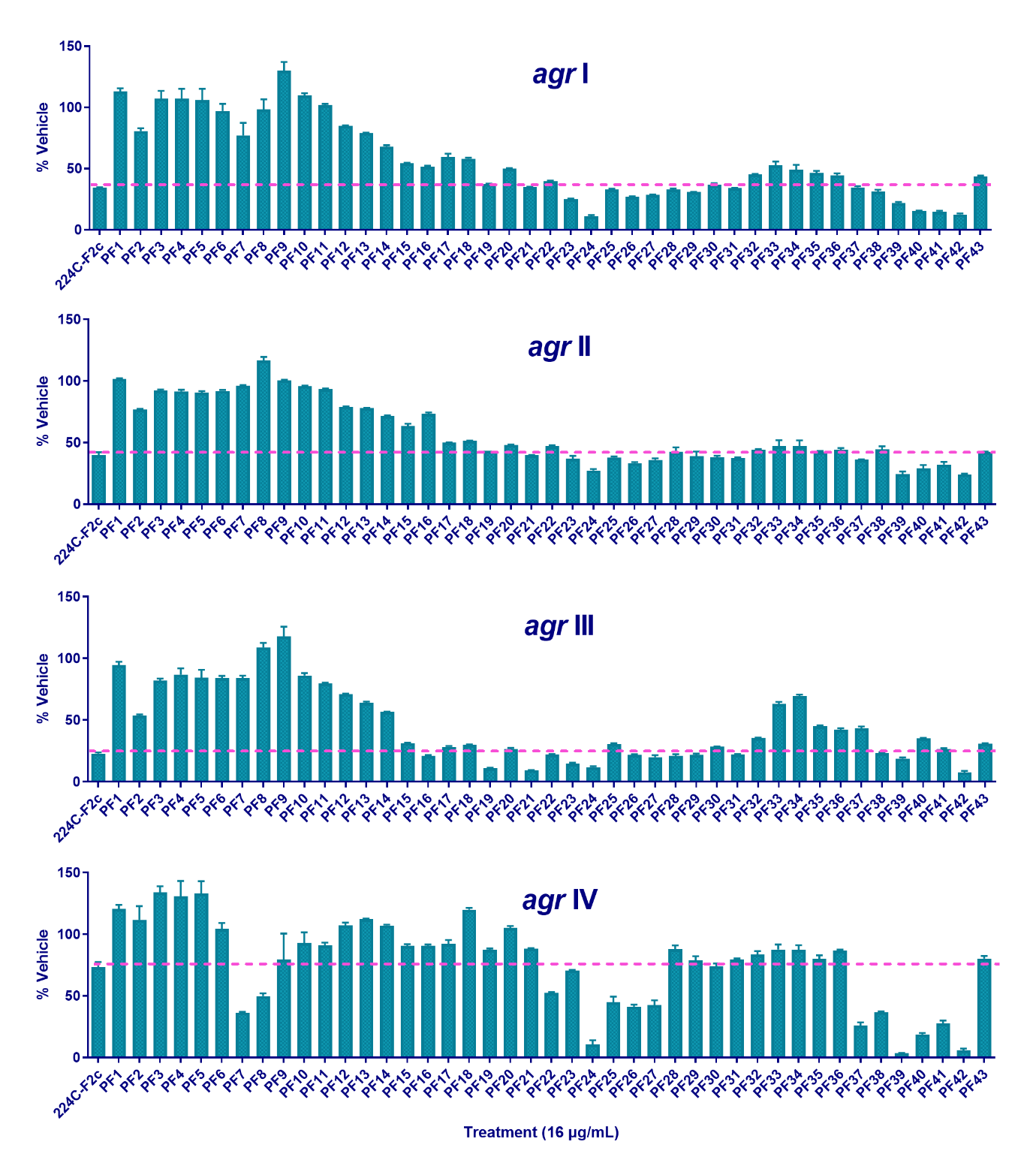


**Figure S2.** Reporter strain screen of *agr*::P3 expression indicates fraction 224C-F2c and 224C-F2c-PF42 are potential sources of quorum sensing inhibitors. *S. aureus* reporter strains of AIP-induced *agr*::P3 activation representing the four *agr* subtypes were used to screen the fractions of 224C-F2c at 16 µg/mL. The pink dashed lines indicate the level of activity of the parent fraction, 224C-F2c. None of the treatments affected optical density of culture at the test concertations. Subtypes of *agr* represented by each graph are shown. All assays were performed in duplicate. The following MRSA strains were used: AH1677 (*agr* I)*,* AH430 (*agr* II)*,* AH1747 (*agr* III), and AH1872 (*agr* IV)*.*





| Peak mass | Display formula | RDB | Delta [ppm] | Theoretical mass | Combined Score | MS Cov. [%] |
| --- | --- | --- | --- | --- | --- | --- |
| 503.33809 | C_30_H_47_0_6_ | 7.50 | 2.72 | 503.33672 | 89.45 | 92.54 |

Figure S3. ESI-MS negative mode spectrum and empirical formula calculations of compound **1**.

**Figure S4.** ^13^C NMR spectrum of **1** in CD_3_OD.

**Figure S5.** DEPT-135 spectrum of **1** in CD_3_OD.

**Figure S6.** HSQC spectrum of **1** in CD_3_OD.

**Figure S7.** ^1^H NMR spectrum of **1** in CD_3_OD.

**Figure S8.** COSY spectrum of **1** in CD_3_OD.

**Figure S9.** HMBC spectrum of **1** in CD_3_OD.

**Figure S10.** NOESY spectrum of **1** in CD_3_OD.





| Peak mass | Display formula | RDB | Delta [ppm] | Theoretical mass | Combined Score | MS Cov. [%] |
| --- | --- | --- | --- | --- | --- | --- |
| 503.33918 | C_30_H_47_0_6_ | 7.50 | 4.90 | 503.33672 | 82.78 | 86.61 |

**Figure S11.** ESI-MS negative mode spectrum and empirical formula calculations of compounds **2a, 2b**.

**Figure S12.** ^1^H NMR spectrum of compounds **2a, 2b** in CD_3_OD.

**Figure S13.** ^13^C NMR spectrum of compounds **2a, 2b** in CD_3_OD.

**Figure S14.** HSQC spectrum of compounds **2a, 2b** in CD_3_OD.

**Figure S15.** COSY spectrum of compounds **2a, 2b** in CD_3_OD.

**Figure S16.** HMBC spectrum of compounds **2a, 2b** in CD_3_OD.

**Figure S17.** Zoomed in ^1^H spectrum of 224C-F2c-PF42. The triplet signal δ_H_ 5.10 ppm is suggested to correspond to H_1_-24 of the proposed castaneroxy precursor, reported by Popova, *et al* **(Popova et al., 2009)**.


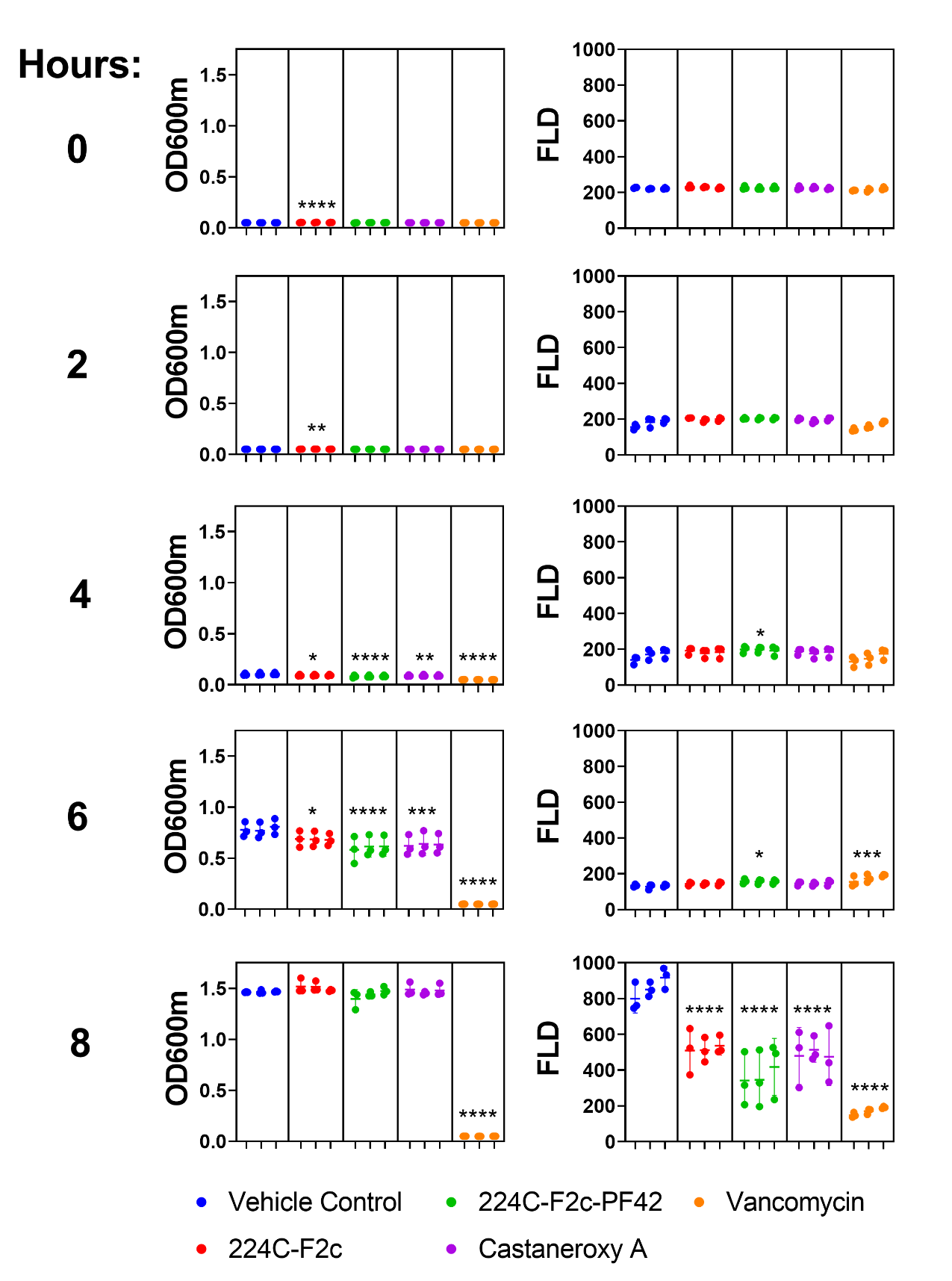


**Figure S18.** Effects of 2 μg/mL castaneroxy A and parent fractions on AH1677 over 8 h. Optical density (OD_600_, left panels) and fluorescence (FLD, right panels) were tracked for the *agr* I reporter AH1677 over an 8-h period at a test concentration of 2 μg/mL, with readings taken every 2 hours. Each experiment included three technical replicates, and three experiments were conducted on separate days for each drug-dose combination (n=9), with all values plotted. Statistical significance was determined by nested one-way ANOVA in comparison to vehicle control. P-values: *: P<0.05, ** P<0.01, ***: P<0.001, ****: P<0.0001.


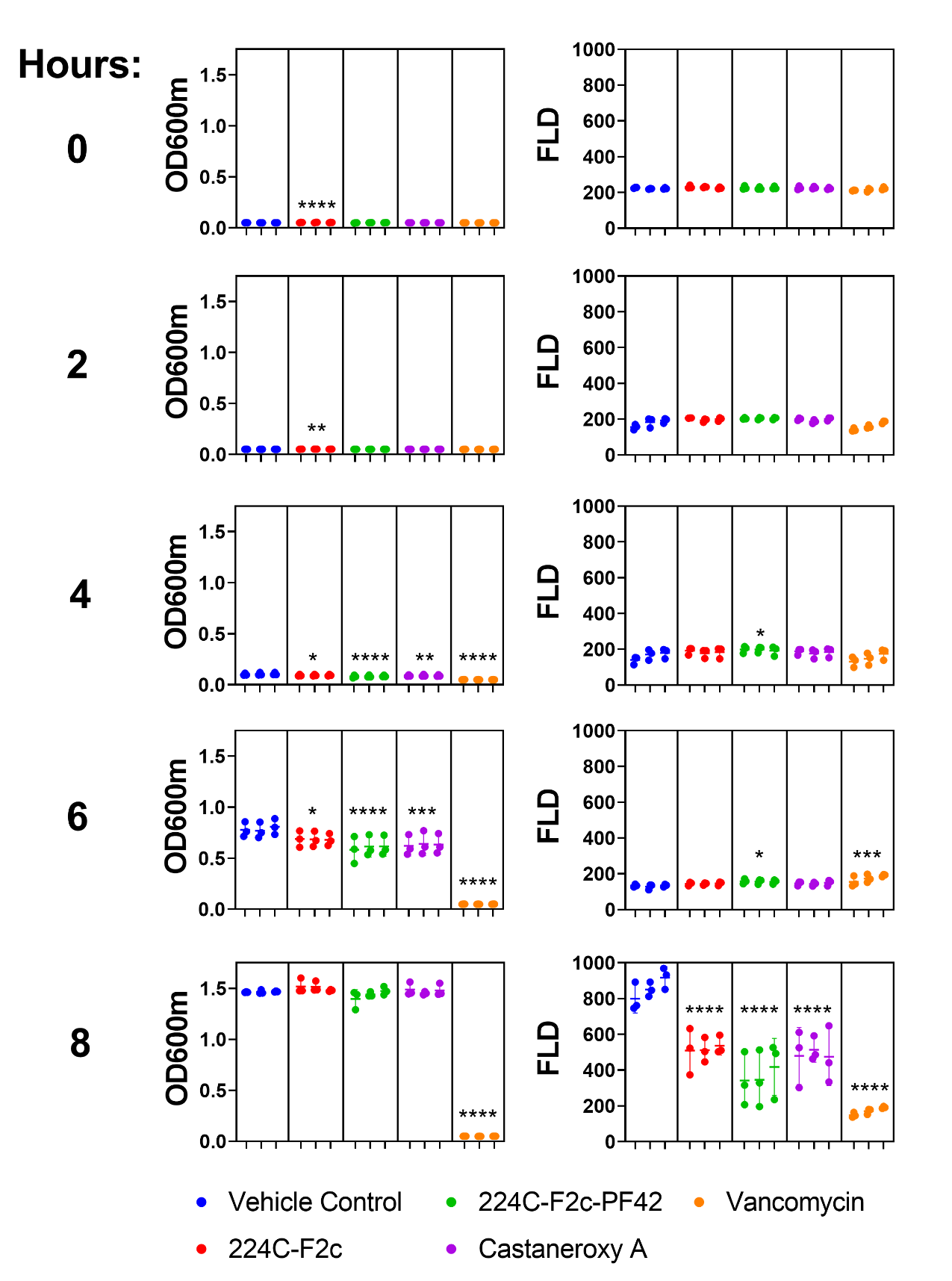


**Figure S19.** Effects of 4 μg/mL castaneroxy A and parent fractions on AH1677 over 8 h. Optical density (OD_600_, left panels) and fluorescence (FLD, right panels) were tracked for the *agr* I reporter AH1677 over an 8-h period at a test concentration of 4 μg/mL, with readings taken every 2 hours. Each experiment included three technical replicates, and three experiments were conducted on separate days for each drug-dose combination (n=9), with all values plotted. Statistical significance was determined by nested one-way ANOVA in comparison to vehicle control. P-values: *: P<0.05, ** P<0.01, ***: P<0.001, ****: P<0.0001.


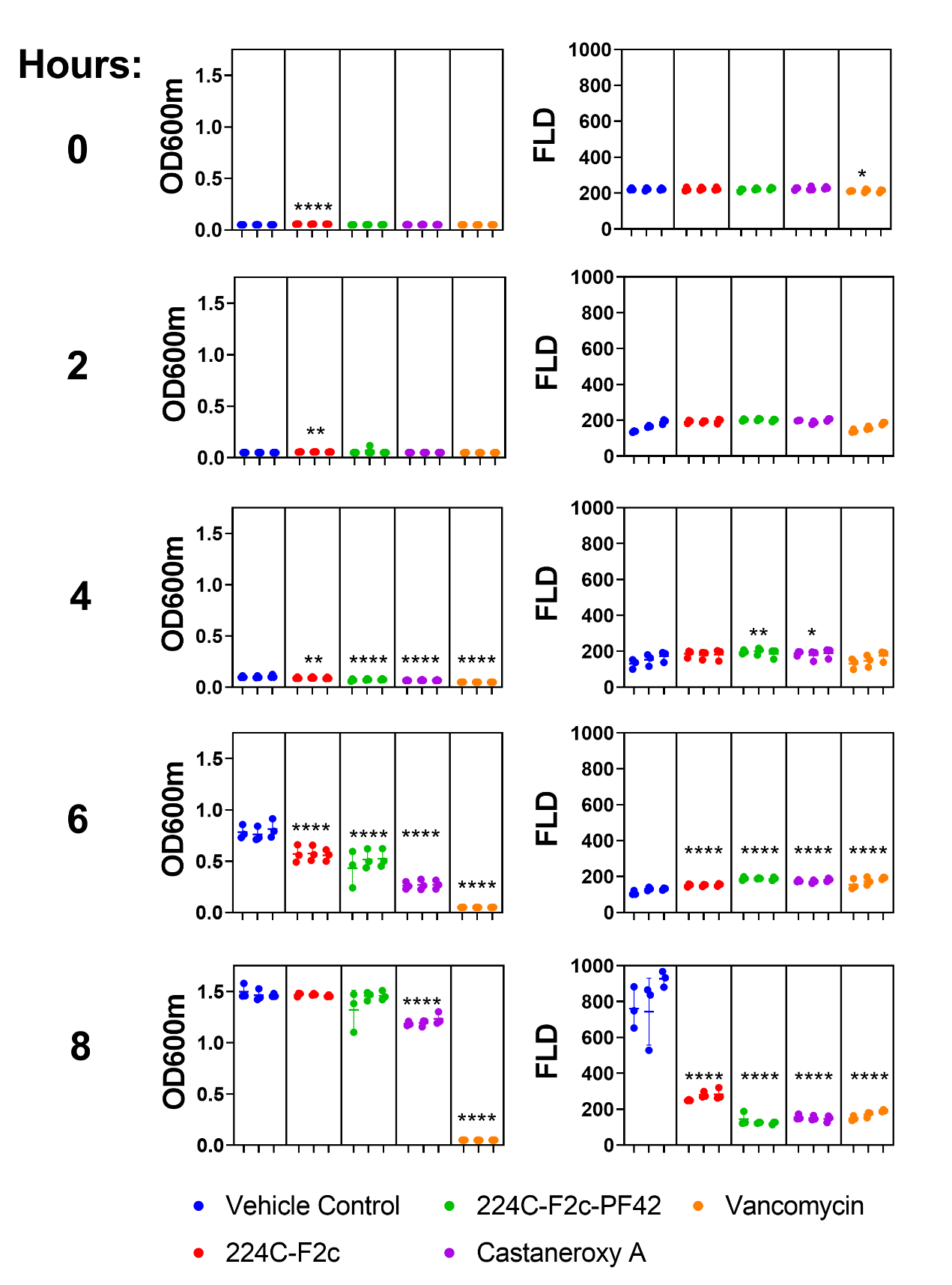


**Figure S20.** Effects of 8 μg/mL castaneroxy A and parent fractions on AH1677 over 8 h. Optical density (OD_600_, left panels) and fluorescence (FLD, right panels) were tracked for the *agr* I reporter AH1677 over an 8-h period at a test concentration of 8 μg/mL, with readings taken every 2 hours. Each experiment included three technical replicates, and three experiments were conducted on separate days for each drug-dose combination (n=9), with all values plotted. Statistical significance was determined by nested one-way ANOVA in comparison to vehicle control. P-values: *: P<0.05, ** P<0.01, ***: P<0.001, ****: P<0.0001.


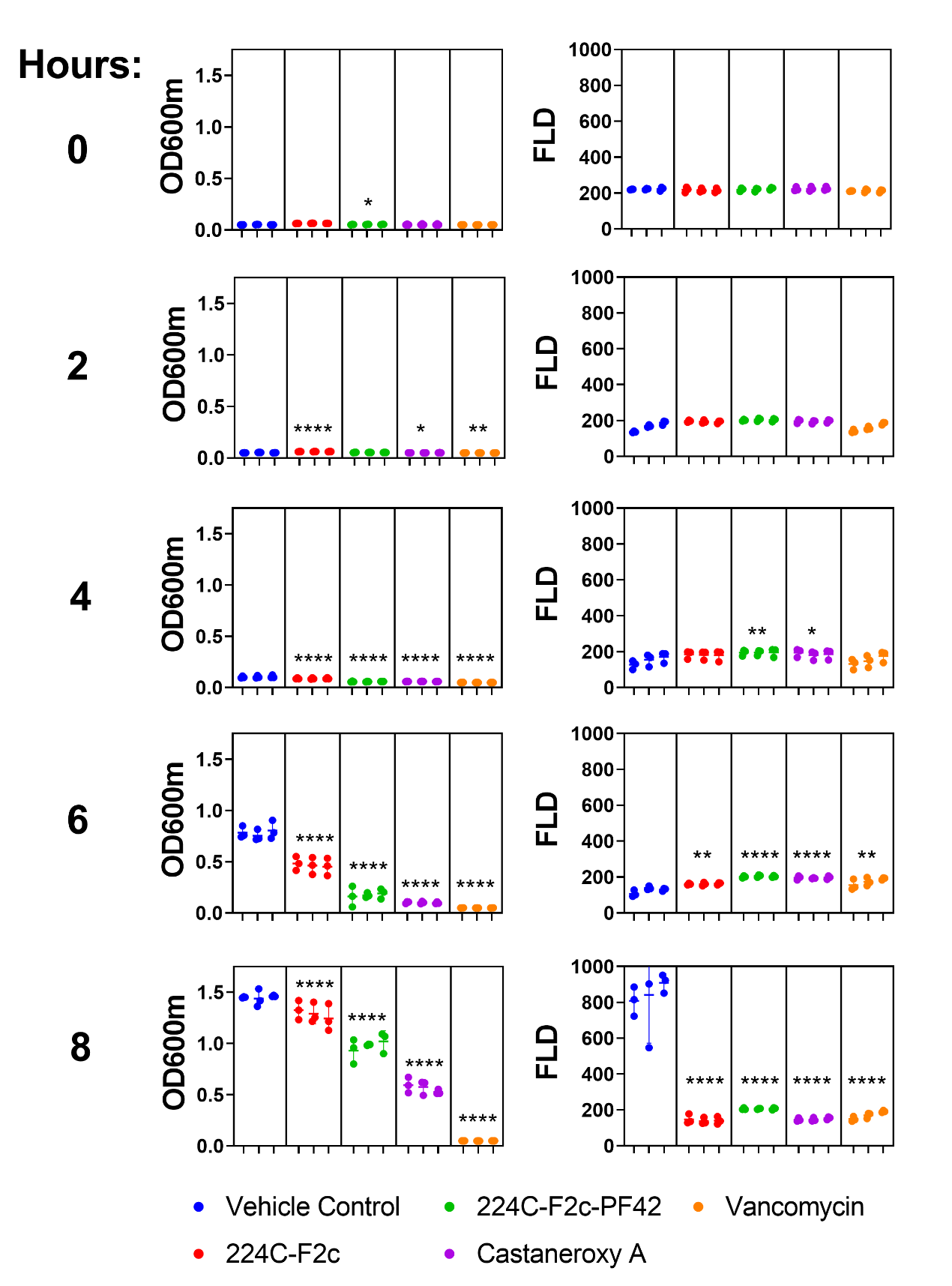


**Figure S21.** Effects of 16 μg/mL castaneroxy A and parent fractions on AH1677 over 8 h. Optical density (OD_600_, left panels) and fluorescence (FLD, right panels) were tracked for the *agr* I reporter AH1677 over an 8-h period at a test concentration of 16 μg/mL, with readings taken every 2 hours. Each experiment included three technical replicates, and three experiments were conducted on separate days for each drug-dose combination (n=9), with all values plotted. Statistical significance was determined by nested one-way ANOVA in comparison to vehicle control. P-values: *: P<0.05, ** P<0.01, ***: P<0.001, ****: P<0.0001.


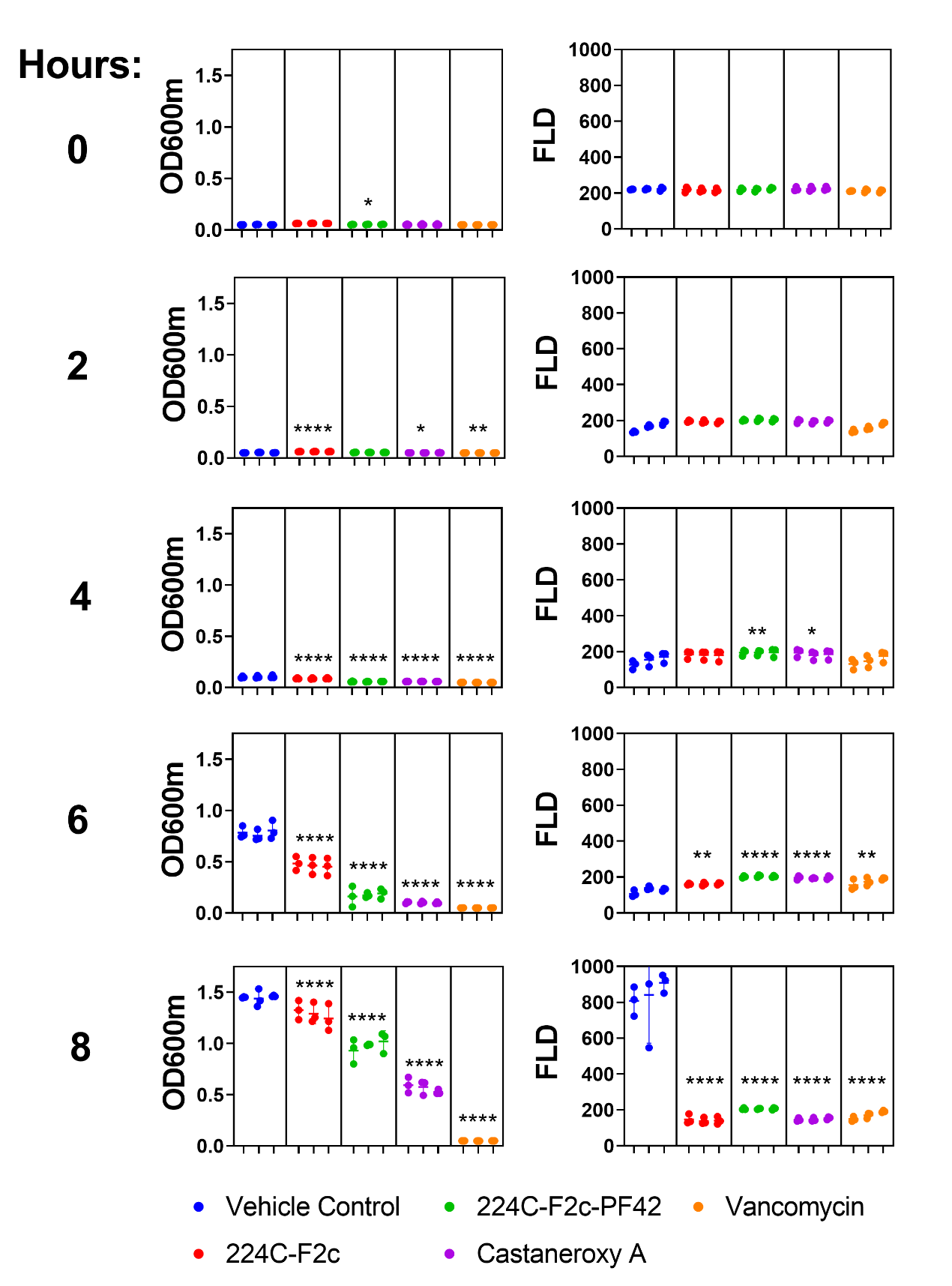


**Figure S22.** Effects of 32 μg/mL castaneroxy A and parent fractions on AH1677 over 8 h. Optical density (OD_600_, left panels) and fluorescence (FLD, right panels) were tracked for the *agr* I reporter AH1677 over an 8-h period at a test concentration of 32 μg/mL, with readings taken every 2 hours. Each experiment included three technical replicates, and three experiments were conducted on separate days for each drug-dose combination (n=9), with all values plotted. Statistical significance was determined by nested one-way ANOVA in comparison to vehicle control. P-values: *: P<0.05, ** P<0.01, ***: P<0.001, ****: P<0.0001.

## Supplementary Tables

**Table S1.** Profiles of *S. aureus* strains used in this study.

| **Strain ID** | **Other Characteristics** | **Ref.** |
| --- | --- | --- |
| AH1677 | AH845 + pDB59 Cm^R^, yfp reporter, *agr* type I | (Kirchdoerfer et al., 2011) |
| AH430 | SA502a + pDB59 Cm^R^, yfp reporter, *agr* type II | (Kirchdoerfer et al., 2011) |
| AH1747 | MW2 + pDB59 Cm^R^, yfp reporter, *agr* type III | (Kirchdoerfer et al., 2011) |
| AH1872 | MN EV(407) + pDB59 Cm^R^, yfp reporter, *agr* type IV | (Kirchdoerfer et al., 2011) |
| AH1263 (LAC) | CA-MRSA, PFT USA300, *agr* type I | (Boles et al., 2010) |
| AH1589 | *hla::Tn551* mutant of AH1263 | (Olson et al., 2013) |
| AH1292 | *Δagr::tetM* mutant of AH1263 | (Olson et al., 2013) |
| NRS385 | HA-MRSA, PFT USA500, MLST ST8, SCC mecIV, sea+, seb+, *agr* type I | (McDougal et al., 2003) |
| NRS249 | sea+, (lukE-lukD)+, hlgv+, associated with native valve endocarditis; SCCmec type IV, agr type I |  |
| UAMS-1 | MSSA, osteomyelitis isolate, strong biofilm producer | (Gillaspy et al., 1995) |
| UAMS-929 | Isogenic *sarA* mutant of UAMS-1, biofilm production is deficient | (Beenken et al., 2004) |

**Table S2.** Comparison of select activities of other *S. aureus* quorum sensing inhibitors.

| **Compound name** | **Assay** | **IC** | **Value (µM)** | **Ref.** |
| --- | --- | --- | --- | --- |
| 3-oxo-olean-12-en-28-oic acid | *agr*::P3 reporter panel | IC_50_ | 4-64 | (Tang et al., 2020) |
| Apicidin | *agr*::P3 reporter panel | IC_50_ | 6.3-50 | (Parlet et al., 2019) |
| ω-Hydroxyemodin | *agr*::P3 reporter panel | IC_50_ | 25-50 | (Daly et al., 2015) |
| Savirin | Rabbit erythrocyte hemolysis | IC_90_ | 13.6 | (Sully et al., 2014) |
| PLNA34 | Rabbit erythrocyte hemolysis | IC_90_ | 12.5 | (Tan et al., 2018) |
| Ambuic acid | LAC AIP inhibition | IC_50_ | 2.5 | (Todd et al., 2017) |

# References

Beenken, K.E., Dunman, P.M., Mcaleese, F., Macapagal, D., Murphy, E., Projan, S.J., Blevins, J.S., and Smeltzer, M.S. (2004). Global Gene Expression in *Staphylococcus aureus* Biofilms. *J. Bacteriol.* 186**,** 4665-4684.

Boles, B.R., Thoendel, M., Roth, A.J., and Horswill, A.R. (2010). Identification of genes involved in polysaccharide-independent *Staphylococcus aureus* biofilm formation. *PLoS One* 5**,** e10146.

Daly, S.M., Elmore, B.O., Kavanaugh, J.S., Triplett, K.D., Figueroa, M., Raja, H.A., El-Elimat, T., Crosby, H.A., Femling, J.K., Cech, N.B., Horswill, A.R., Oberlies, N.H., and Hall, P.R. (2015). ω-Hydroxyemodin limits *Staphylococcus aureus* quorum sensing-mediated pathogenesis and inflammation. *Antimicrob. Agents Chemother.* 59**,** 2223-2235.

Gillaspy, A.F., Hickmon, S.G., Skinner, R.A., Thomas, J.R., Nelson, C.L., and Smeltzer, M.S. (1995). Role of the accessory gene regulator (agr) in pathogenesis of staphylococcal osteomyelitis. *Infect. Immun.* 63**,** 3373-3380.

Kirchdoerfer, R.N., Garner, A.L., Flack, C.E., Mee, J.M., Horswill, A.R., Janda, K.D., Kaufmann, G.F., and Wilson, I.A. (2011). Structural basis for ligand recognition and discrimination of a quorum-quenching antibody. *J. Biol. Chem.* 286**,** 17351-17358.

Mcdougal, L.K., Steward, C.D., Killgore, G.E., Chaitram, J.M., Mcallister, S.K., and Tenover, F.C. (2003). Pulsed-Field Gel Electrophoresis Typing of Oxacillin-Resistant <em>Staphylococcus aureus</em> Isolates from the United States: Establishing a National Database. *Journal of Clinical Microbiology* 41**,** 5113-5120.

Olson, M.E., Nygaard, T.K., Ackermann, L., Watkins, R.L., Zurek, O.W., Pallister, K.B., Griffith, S., Kiedrowski, M.R., Flack, C.E., Kavanaugh, J.S., Kreiswirth, B.N., Horswill, A.R., and Voyich, J.M. (2013). Staphylococcus aureus nuclease is an SaeRS-dependent virulence factor. *Infect. Immun.* 81**,** 1316-1324.

Parlet, C.P., Kavanaugh, J.S., Crosby, H.A., Raja, H.A., El-Elimat, T., Todd, D.A., Pearce, C.J., Cech, N.B., Oberlies, N.H., and Horswill, A.R. (2019). Apicidin attenuates MRSA virulence through quorum-sensing inhibition and enhanced host defense. *Cell Rep.* 27**,** 187-198.e186.

Popova, M.P., Chinou, I.B., Marekov, I.N., and Bankova, V.S. (2009). Terpenes with antimicrobial activity from *Cretan propolis*. *Phytochemistry* 70**,** 1262-1271.

Sully, E.K., Malachowa, N., Elmore, B.O., Alexander, S.M., Femling, J.K., Gray, B.M., Deleo, F.R., Otto, M., Cheung, A.L., Edwards, B.S., Sklar, L.A., Horswill, A.R., Hall, P.R., and Gresham, H.D. (2014). Selective chemical inhibition of *agr* quorum sensing in *Staphylococcus aureus* promotes host defense with minimal impact on resistance. *PLoS Pathog.* 10**,** e1004174.

Tan, L., Li, S.R., Jiang, B., Hu, X.M., and Li, S. (2018). Therapeutic targeting of the *Staphylococcus aureus* accessory gene regulator (*agr*) system. *Front. Microbiol.* 9**,** 55.

Tang, H., Porras, G., Brown, M.M., Chassagne, F., Lyles, J.T., Bacsa, J., Horswill, A.R., and Quave, C.L. (2020). Triterpenoid acids isolated from *Schinus terebinthifolia* fruits reduce *Staphylococcus aureus* virulence and abate dermonecrosis. *Sci. Rep.* 10**,** 8046.

Todd, D.A., Parlet, C.P., Crosby, H.A., Malone, C.L., Heilmann, K.P., Horswill, A.R., and Cech, N.B. (2017). Signal biosynthesis inhibition with ambuic acid as a strategy to target antibiotic-resistant infections. *Antimicrob. Agents Chemother.* 61**,** e00263-00217.
