# Supplementary material for: Castaneroxy A From the Leaves of Castanea sativa Inhibits Virulence in Staphylococcus aureus
Source: Front Pharmacol. 2021 Jun 28;12:640179. doi: 10.3389/fphar.2021.640179 (PMC8274328; doi:10.3389/fphar.2021.640179)

checkCIF/PLATON report

Structure factors have been supplied for datablock(s) castaneroxy-b

THIS REPORT IS FOR GUIDANCE ONLY. IF USED AS PART OF A REVIEW PROCEDURE FOR PUBLICATION, IT SHOULD NOT REPLACE THE EXPERTISE OF AN EXPERIENCED CRYSTALLOGRAPHIC REFEREE.

No syntax errors found.   CIF dictionary   Interpreting this report

Datablock: castaneroxy-b

```
Bond precision:  C-C = 0.0068 A          Wavelength=1.54184

Cell:           a=6.7511(3)    b=12.2548(7)       c=17.4683(12)
                alpha=90       beta=98.733(6)     gamma=90
Temperature:    110 K

               Calculated                 Reported
Volume         1428.46(14)                1428.45(15)
Space group    P 21                       P 1 21 1
Hall group     P 2yb                      P 2yb
Moiety formula C30 H48 O6, 2(H2 O)        2(C30 H48 O6), 4(H2 O)
Sum formula    C30 H52 O8                 C60 H104 O16
Mr             540.72                     1081.43
Dx,g cm-3      1.257                      1.257
Z              2                          1
Mu (mm-1)      0.723                      0.723
F000           592.0                      592.0
F000'          593.81
h,k,lmax       7,14,20                    7,14,20
Nref           4859[ 2553]                3583
Tmin,Tmax      0.815,0.927                0.354,1.000
Tmin'          0.702

Correction method= # Reported T Limits: Tmin=0.354 Tmax=1.000
AbsCorr = GAUSSIAN

Data completeness= 1.40/0.74      Theta(max)= 65.072

R(reflections)= 0.0610( 2898)     wR2(reflections)= 0.1641( 3583)

S = 1.005                  Npar= 364
```

```
The following ALERTS were generated. Each ALERT has the format
       test-name_ALERT_alert-type_alert-level.
Click on the hyperlinks for more details of the test.
```

Alert level B

```
PLAT355_ALERT_3_B Long   O-H (X0.82,N0.98A)  O6       - H6       .       1.09 Ang.
```

Author Response: This was a freely refined hydrogen atom. It is long because it was refined without restraints.

```
PLAT410_ALERT_2_B Short Intra H...H Contact  H1B      ..H11B     .       1.88 Ang.
                                                      x,y,z  =      1_555 Check
```

Author Response: This distance is real and is the distance between two hydrogens of a fused ring system.

```
PLAT915_ALERT_3_B No Flack x Check Done: Low Friedel Pair Coverage         46 %
```

Author Response: Because the Friedel Pair Coverage was low the absolute configuration was assigned using the known absolute configuration of the partner molecule, castaneroxy-a.

Alert level C

```
STRVA01_ALERT_4_C           Flack test results are ambiguous.
           From the CIF: _refine_ls_abs_structure_Flack    0.500
           From the CIF: _refine_ls_abs_structure_Flack_su    0.400
THETM01_ALERT_3_C  The value of sine(theta_max)/wavelength is less than 0.590
            Calculated sin(theta_max)/wavelength =    0.5882
PLAT089_ALERT_3_C Poor Data / Parameter Ratio (Zmax < 18) ........       6.90 Note
PLAT340_ALERT_3_C Low Bond Precision on  C-C Bonds ...............    0.00679 Ang.
PLAT351_ALERT_3_C Long   C-H (X0.96,N1.08A)  C1       - H1A      .       1.13 Ang.
PLAT351_ALERT_3_C Long   C-H (X0.96,N1.08A)  C1       - H1B      .       1.12 Ang.
PLAT351_ALERT_3_C Long   C-H (X0.96,N1.08A)  C2       - H2A      .       1.12 Ang.
PLAT351_ALERT_3_C Long   C-H (X0.96,N1.08A)  C2       - H2B      .       1.12 Ang.
PLAT351_ALERT_3_C Long   C-H (X0.96,N1.08A)  C5       - H5       .       1.11 Ang.
PLAT351_ALERT_3_C Long   C-H (X0.96,N1.08A)  C6       - H6A      .       1.12 Ang.
PLAT351_ALERT_3_C Long   C-H (X0.96,N1.08A)  C6       - H6B      .       1.12 Ang.
PLAT351_ALERT_3_C Long   C-H (X0.96,N1.08A)  C11      - H11A     .       1.12 Ang.
PLAT351_ALERT_3_C Long   C-H (X0.96,N1.08A)  C11      - H11B     .       1.12 Ang.
PLAT351_ALERT_3_C Long   C-H (X0.96,N1.08A)  C12      - H12A     .       1.12 Ang.
PLAT351_ALERT_3_C Long   C-H (X0.96,N1.08A)  C12      - H12B     .       1.12 Ang.
PLAT351_ALERT_3_C Long   C-H (X0.96,N1.08A)  C15      - H15A     .       1.12 Ang.
PLAT351_ALERT_3_C Long   C-H (X0.96,N1.08A)  C15      - H15B     .       1.12 Ang.
PLAT351_ALERT_3_C Long   C-H (X0.96,N1.08A)  C16      - H16A     .       1.12 Ang.
PLAT351_ALERT_3_C Long   C-H (X0.96,N1.08A)  C16      - H16B     .       1.12 Ang.
PLAT351_ALERT_3_C Long   C-H (X0.96,N1.08A)  C19      - H19A     .       1.12 Ang.
PLAT351_ALERT_3_C Long   C-H (X0.96,N1.08A)  C19      - H19B     .       1.13 Ang.
PLAT351_ALERT_3_C Long   C-H (X0.96,N1.08A)  C20      - H20      .       1.12 Ang.
PLAT351_ALERT_3_C Long   C-H (X0.96,N1.08A)  C22      - H22A     .       1.12 Ang.
PLAT351_ALERT_3_C Long   C-H (X0.96,N1.08A)  C22      - H22B     .       1.12 Ang.
PLAT351_ALERT_3_C Long   C-H (X0.96,N1.08A)  C23      - H23      .       1.11 Ang.
PLAT351_ALERT_3_C Long   C-H (X0.96,N1.08A)  C24      - H24      .       1.16 Ang.
PLAT355_ALERT_3_C Long   O-H (X0.82,N0.98A)  O1W      - H1WA     .       1.01 Ang.
```

Author Response: This was a freely refined hydrogen atom. It is long because it was refined without restraints.

```
PLAT355_ALERT_3_C Long   O-H (X0.82,N0.98A)  O1W      - H1WB     .       1.01 Ang.
```

Author Response: This was a freely refined hydrogen atom. It is long because it was refined without restraints.

```
PLAT355_ALERT_3_C Long   O-H (X0.82,N0.98A)  O2W      - H2WA     .       1.01 Ang.
```

Author Response: This was a freely refined hydrogen atom. It is long because it was refined without restraints.

```
PLAT355_ALERT_3_C Long   O-H (X0.82,N0.98A)  O2W      - H2WB     .       1.01 Ang.
```

Author Response: This was a freely refined hydrogen atom. It is long because it was refined without restraints.

```
PLAT751_ALERT_4_C Bond    Calc     0.97000, Rep    0.968(3) ......  Senseless s.u.
              O3       -H3            1.555   1.555 ........     #    2 Check
PLAT751_ALERT_4_C Bond    Calc     0.97000, Rep    0.966(3) ......  Senseless s.u.
              O1       -H1            1.555   1.555 ........     #    4 Check
PLAT751_ALERT_4_C Bond    Calc     0.97000, Rep    0.965(3) ......  Senseless s.u.
              O4       -H4            1.555   1.555 ........     #    6 Check
PLAT751_ALERT_4_C Bond    Calc     1.09000, Rep    1.085(4) ......  Senseless s.u.
              O6       -H6            1.555   1.555 ........     #   10 Check
PLAT751_ALERT_4_C Bond    Calc     1.12000, Rep    1.123(3) ......  Senseless s.u.
              C6       -H6A           1.555   1.555 ........     #   18 Check
PLAT751_ALERT_4_C Bond    Calc     1.12000, Rep    1.125(3) ......  Senseless s.u.
              C6       -H6B           1.555   1.555 ........     #   19 Check
PLAT751_ALERT_4_C Bond    Calc     1.06000, Rep    1.058(5) ......  Senseless s.u.
              C7       -H7            1.555   1.555 ........     #   21 Check
PLAT751_ALERT_4_C Bond    Calc     1.12000, Rep    1.123(3) ......  Senseless s.u.
              C15      -H15A          1.555   1.555 ........     #   29 Check
PLAT751_ALERT_4_C Bond    Calc     1.12000, Rep    1.125(3) ......  Senseless s.u.
              C15      -H15B          1.555   1.555 ........     #   30 Check
PLAT751_ALERT_4_C Bond    Calc     1.12000, Rep    1.124(3) ......  Senseless s.u.
              C16      -H16A          1.555   1.555 ........     #   32 Check
PLAT751_ALERT_4_C Bond    Calc     1.12000, Rep    1.123(3) ......  Senseless s.u.
              C16      -H16B          1.555   1.555 ........     #   33 Check
PLAT751_ALERT_4_C Bond    Calc     0.93000, Rep    0.932(5) ......  Senseless s.u.
              C17      -H17           1.555   1.555 ........     #   35 Check
PLAT751_ALERT_4_C Bond    Calc     1.12000, Rep    1.124(3) ......  Senseless s.u.
              C12      -H12A          1.555   1.555 ........     #   40 Check
PLAT751_ALERT_4_C Bond    Calc     1.12000, Rep    1.124(3) ......  Senseless s.u.
              C12      -H12B          1.555   1.555 ........     #   41 Check
PLAT751_ALERT_4_C Bond    Calc     1.12000, Rep    1.123(3) ......  Senseless s.u.
              C11      -H11A          1.555   1.555 ........     #   43 Check
PLAT751_ALERT_4_C Bond    Calc     1.12000, Rep    1.123(3) ......  Senseless s.u.
              C11      -H11B          1.555   1.555 ........     #   44 Check
PLAT751_ALERT_4_C Bond    Calc     1.12000, Rep    1.124(2) ......  Senseless s.u.
              C19      -H19A          1.555   1.555 ........     #   47 Check
PLAT751_ALERT_4_C Bond    Calc     1.13000, Rep    1.125(2) ......  Senseless s.u.
              C19      -H19B          1.555   1.555 ........     #   48 Check
PLAT751_ALERT_4_C Bond    Calc     1.00000, Rep    1.005(5) ......  Senseless s.u.
              C3       -H3A           1.555   1.555 ........     #   52 Check
```

```
PLAT751_ALERT_4_C Bond    Calc     1.12000, Rep    1.123(3) ......  Senseless s.u.
              C2       -H2A            1.555   1.555 ........     #   54 Check
PLAT751_ALERT_4_C Bond    Calc     1.12000, Rep    1.123(3) ......  Senseless s.u.
              C2       -H2B            1.555   1.555 ........     #   55 Check
PLAT751_ALERT_4_C Bond    Calc     1.13000, Rep    1.126(3) ......  Senseless s.u.
              C1       -H1A            1.555   1.555 ........     #   57 Check
PLAT751_ALERT_4_C Bond    Calc     1.12000, Rep    1.124(3) ......  Senseless s.u.
              C1       -H1B            1.555   1.555 ........     #   58 Check
PLAT751_ALERT_4_C Bond    Calc     1.12000, Rep    1.124(3) ......  Senseless s.u.
              C20      -H20            1.555   1.555 ........     #   62 Check
PLAT751_ALERT_4_C Bond    Calc     1.12000, Rep    1.123(3) ......  Senseless s.u.
              C22      -H22A           1.555   1.555 ........     #   65 Check
PLAT751_ALERT_4_C Bond    Calc     1.12000, Rep    1.124(3) ......  Senseless s.u.
              C22      -H22B           1.555   1.555 ........     #   66 Check
PLAT751_ALERT_4_C Bond    Calc     1.11000, Rep    1.106(5) ......  Senseless s.u.
              C23      -H23            1.555   1.555 ........     #   68 Check
PLAT751_ALERT_4_C Bond    Calc     1.05000, Rep    1.051(4) ......  Senseless s.u.
              C27      -H27A           1.555   1.555 ........     #   74 Check
PLAT751_ALERT_4_C Bond    Calc     1.05000, Rep    1.052(4) ......  Senseless s.u.
              C27      -H27B           1.555   1.555 ........     #   75 Check
PLAT751_ALERT_4_C Bond    Calc     1.05000, Rep    1.050(4) ......  Senseless s.u.
              C27      -H27C           1.555   1.555 ........     #   76 Check
PLAT751_ALERT_4_C Bond    Calc     1.05000, Rep    1.054(4) ......  Senseless s.u.
              C26      -H26A           1.555   1.555 ........     #   77 Check
PLAT751_ALERT_4_C Bond    Calc     1.05000, Rep    1.052(3) ......  Senseless s.u.
              C26      -H26B           1.555   1.555 ........     #   78 Check
PLAT751_ALERT_4_C Bond    Calc     1.05000, Rep    1.051(4) ......  Senseless s.u.
              C26      -H26C           1.555   1.555 ........     #   79 Check
PLAT752_ALERT_4_C Angle   Calc      109.00, Rep    108.5(4) ......  Senseless s.u.
              C28     -O3      -H3        1.555  1.555  1.555     #    1 Check
PLAT752_ALERT_4_C Angle   Calc      109.00, Rep    108.8(4) ......  Senseless s.u.
              C3      -O1      -H1        1.555  1.555  1.555     #    2 Check
PLAT752_ALERT_4_C Angle   Calc      106.00, Rep    105.6(3) ......  Senseless s.u.
              C7      -O4      -H4        1.555  1.555  1.555     #    3 Check
PLAT752_ALERT_4_C Angle   Calc      103.00, Rep    103.3(4) ......  Senseless s.u.
              O5      -O6      -H6        1.555  1.555  1.555     #    5 Check
PLAT752_ALERT_4_C Angle   Calc      114.00, Rep    113.7(4) ......  Senseless s.u.
              C5      -C6      -H6A       1.555  1.555  1.555     #   18 Check
PLAT752_ALERT_4_C Angle   Calc      103.00, Rep    103.1(3) ......  Senseless s.u.
              C5      -C6      -H6B       1.555  1.555  1.555     #   19 Check
PLAT752_ALERT_4_C Angle   Calc      112.00, Rep    111.7(3) ......  Senseless s.u.
              H6A     -C6      -H6B       1.555  1.555  1.555     #   20 Check
PLAT752_ALERT_4_C Angle   Calc      102.00, Rep    101.8(3) ......  Senseless s.u.
              C7      -C6      -H6A       1.555  1.555  1.555     #   22 Check
PLAT752_ALERT_4_C Angle   Calc      118.00, Rep    118.2(4) ......  Senseless s.u.
              C7      -C6      -H6B       1.555  1.555  1.555     #   23 Check
PLAT752_ALERT_4_C Angle   Calc      102.00, Rep    102.3(4) ......  Senseless s.u.
              O4      -C7      -H7        1.555  1.555  1.555     #   25 Check
PLAT752_ALERT_4_C Angle   Calc      113.00, Rep    112.9(4) ......  Senseless s.u.
              C6      -C7      -H7        1.555  1.555  1.555     #   27 Check
PLAT752_ALERT_4_C Angle   Calc      112.00, Rep    112.0(4) ......  Senseless s.u.
              C8      -C7      -H7        1.555  1.555  1.555     #   29 Check
PLAT752_ALERT_4_C Angle   Calc      112.00, Rep    112.0(4) ......  Senseless s.u.
              C14     -C15     -H15A      1.555  1.555  1.555     #   42 Check
PLAT752_ALERT_4_C Angle   Calc      107.00, Rep    107.1(4) ......  Senseless s.u.
              C14     -C15     -H15B      1.555  1.555  1.555     #   43 Check
PLAT752_ALERT_4_C Angle   Calc       99.00, Rep     98.7(2) ......  Senseless s.u.
              H15A    -C15     -H15B      1.555  1.555  1.555     #   45 Check
PLAT752_ALERT_4_C Angle   Calc      118.00, Rep    118.0(3) ......  Senseless s.u.
              C16     -C15     -H15A      1.555  1.555  1.555     #   46 Check
PLAT752_ALERT_4_C Angle   Calc      116.00, Rep    115.7(3) ......  Senseless s.u.
              C16     -C15     -H15B      1.555  1.555  1.555     #   47 Check
```

```
PLAT752_ALERT_4_C Angle   Calc      112.00, Rep    111.6(3) ......  Senseless s.u.
              C15     -C16     -H16A       1.555  1.555  1.555     #   48 Check
PLAT752_ALERT_4_C Angle   Calc      108.00, Rep    107.5(3) ......  Senseless s.u.
              C15     -C16     -H16B       1.555  1.555  1.555     #   49 Check
PLAT752_ALERT_4_C Angle   Calc      110.00, Rep    109.8(3) ......  Senseless s.u.
              H16A    -C16     -H16B       1.555  1.555  1.555     #   50 Check
PLAT752_ALERT_4_C Angle   Calc      110.00, Rep    109.9(4) ......  Senseless s.u.
              C17     -C16     -H16A       1.555  1.555  1.555     #   52 Check
PLAT752_ALERT_4_C Angle   Calc      111.00, Rep    110.8(4) ......  Senseless s.u.
              C17     -C16     -H16B       1.555  1.555  1.555     #   53 Check
PLAT752_ALERT_4_C Angle   Calc      113.00, Rep    113.4(4) ......  Senseless s.u.
              C16     -C17     -H17        1.555  1.555  1.555     #   54 Check
PLAT752_ALERT_4_C Angle   Calc      104.00, Rep    104.5(4) ......  Senseless s.u.
              C13     -C17     -H17        1.555  1.555  1.555     #   56 Check
PLAT752_ALERT_4_C Angle   Calc      104.00, Rep    103.9(4) ......  Senseless s.u.
              C20     -C17     -H17        1.555  1.555  1.555     #   58 Check
PLAT752_ALERT_4_C Angle   Calc      105.00, Rep    105.3(3) ......  Senseless s.u.
              C13     -C12     -H12A       1.555  1.555  1.555     #   66 Check
PLAT752_ALERT_4_C Angle   Calc      107.00, Rep    107.4(3) ......  Senseless s.u.
              C13     -C12     -H12B       1.555  1.555  1.555     #   67 Check
PLAT752_ALERT_4_C Angle   Calc      111.00, Rep    111.3(3) ......  Senseless s.u.
              H12A    -C12     -H12B       1.555  1.555  1.555     #   69 Check
PLAT752_ALERT_4_C Angle   Calc      110.00, Rep    110.1(3) ......  Senseless s.u.
              C11     -C12     -H12A       1.555  1.555  1.555     #   70 Check
PLAT752_ALERT_4_C Angle   Calc      109.00, Rep    109.3(3) ......  Senseless s.u.
              C11     -C12     -H12B       1.555  1.555  1.555     #   71 Check
PLAT752_ALERT_4_C Angle   Calc      115.00, Rep    114.8(3) ......  Senseless s.u.
              C12     -C11     -H11A       1.555  1.555  1.555     #   72 Check
PLAT752_ALERT_4_C Angle   Calc      113.00, Rep    113.0(3) ......  Senseless s.u.
              C12     -C11     -H11B       1.555  1.555  1.555     #   73 Check
PLAT752_ALERT_4_C Angle   Calc       91.00, Rep   91.03(19) ......  Senseless s.u.
              H11A    -C11     -H11B       1.555  1.555  1.555     #   74 Check
PLAT752_ALERT_4_C Angle   Calc      109.00, Rep    108.9(4) ......  Senseless s.u.
              C9      -C11     -H11A       1.555  1.555  1.555     #   76 Check
PLAT752_ALERT_4_C Angle   Calc      108.00, Rep    107.6(4) ......  Senseless s.u.
              C9      -C11     -H11B       1.555  1.555  1.555     #   77 Check
PLAT752_ALERT_4_C Angle   Calc      115.00, Rep    115.2(3) ......  Senseless s.u.
              C10     -C19     -H19A       1.555  1.555  1.555     #   85 Check
PLAT752_ALERT_4_C Angle   Calc      110.00, Rep    110.3(3) ......  Senseless s.u.
              C10     -C19     -H19B       1.555  1.555  1.555     #   86 Check
PLAT752_ALERT_4_C Angle   Calc      115.00, Rep    114.7(3) ......  Senseless s.u.
              C9      -C19     -H19A       1.555  1.555  1.555     #   87 Check
PLAT752_ALERT_4_C Angle   Calc      119.00, Rep    118.6(3) ......  Senseless s.u.
              C9      -C19     -H19B       1.555  1.555  1.555     #   88 Check
PLAT752_ALERT_4_C Angle   Calc      121.00, Rep    121.6(3) ......  Senseless s.u.
              H19A    -C19     -H19B       1.555  1.555  1.555     #   89 Check
PLAT752_ALERT_4_C Angle   Calc      106.00, Rep    106.2(4) ......  Senseless s.u.
              O1      -C3      -H3A        1.555  1.555  1.555     #   97 Check
PLAT752_ALERT_4_C Angle   Calc       98.00, Rep     98.1(5) ......  Senseless s.u.
              C4      -C3      -H3A        1.555  1.555  1.555     #   99 Check
PLAT752_ALERT_4_C Angle   Calc      118.00, Rep    117.8(5) ......  Senseless s.u.
              C2      -C3      -H3A        1.555  1.555  1.555     #  101 Check
PLAT752_ALERT_4_C Angle   Calc      110.00, Rep    109.5(4) ......  Senseless s.u.
              C3      -C2      -H2A        1.555  1.555  1.555     #  102 Check
PLAT752_ALERT_4_C Angle   Calc      111.00, Rep    110.9(4) ......  Senseless s.u.
              C3      -C2      -H2B        1.555  1.555  1.555     #  103 Check
PLAT752_ALERT_4_C Angle   Calc      106.00, Rep    106.4(3) ......  Senseless s.u.
              H2A     -C2      -H2B        1.555  1.555  1.555     #  105 Check
PLAT752_ALERT_4_C Angle   Calc      114.00, Rep    114.3(4) ......  Senseless s.u.
              C1      -C2      -H2A        1.555  1.555  1.555     #  106 Check
PLAT752_ALERT_4_C Angle   Calc      103.00, Rep    103.5(3) ......  Senseless s.u.
              C1      -C2      -H2B        1.555  1.555  1.555     #  107 Check
```

```
PLAT752_ALERT_4_C Angle   Calc      108.00, Rep    107.6(4) ......  Senseless s.u.
              C10     -C1      -H1A        1.555  1.555  1.555     #  109 Check
PLAT752_ALERT_4_C Angle   Calc      109.00, Rep    109.4(4) ......  Senseless s.u.
              C10     -C1      -H1B        1.555  1.555  1.555     #  110 Check
PLAT752_ALERT_4_C Angle   Calc      108.00, Rep    108.5(3) ......  Senseless s.u.
              C2      -C1      -H1A        1.555  1.555  1.555     #  111 Check
PLAT752_ALERT_4_C Angle   Calc      111.00, Rep    111.2(3) ......  Senseless s.u.
              C2      -C1      -H1B        1.555  1.555  1.555     #  112 Check
PLAT752_ALERT_4_C Angle   Calc      111.00, Rep    110.9(3) ......  Senseless s.u.
              H1A     -C1      -H1B        1.555  1.555  1.555     #  113 Check
PLAT752_ALERT_4_C Angle   Calc      108.00, Rep    107.6(4) ......  Senseless s.u.
              C17     -C20     -H20        1.555  1.555  1.555     #  123 Check
PLAT752_ALERT_4_C Angle   Calc      103.00, Rep    102.9(3) ......  Senseless s.u.
              C22     -C20     -H20        1.555  1.555  1.555     #  126 Check
PLAT752_ALERT_4_C Angle   Calc      114.00, Rep    114.4(4) ......  Senseless s.u.
              C21     -C20     -H20        1.555  1.555  1.555     #  128 Check
PLAT752_ALERT_4_C Angle   Calc      108.00, Rep    107.6(3) ......  Senseless s.u.
              C20     -C22     -H22A       1.555  1.555  1.555     #  129 Check
PLAT752_ALERT_4_C Angle   Calc      106.00, Rep    106.3(4) ......  Senseless s.u.
              C20     -C22     -H22B       1.555  1.555  1.555     #  130 Check
PLAT752_ALERT_4_C Angle   Calc      112.00, Rep    112.4(3) ......  Senseless s.u.
              H22A    -C22     -H22B       1.555  1.555  1.555     #  131 Check
PLAT752_ALERT_4_C Angle   Calc      105.00, Rep    105.0(4) ......  Senseless s.u.
              C23     -C22     -H22A       1.555  1.555  1.555     #  133 Check
PLAT752_ALERT_4_C Angle   Calc      111.00, Rep    111.5(4) ......  Senseless s.u.
              C23     -C22     -H22B       1.555  1.555  1.555     #  134 Check
PLAT752_ALERT_4_C Angle   Calc      108.00, Rep    107.9(4) ......  Senseless s.u.
              C22     -C23     -H23        1.555  1.555  1.555     #  135 Check
PLAT752_ALERT_4_C Angle   Calc      127.00, Rep    127.1(5) ......  Senseless s.u.
              C24     -C23     -H23        1.555  1.555  1.555     #  137 Check
PLAT752_ALERT_4_C Angle   Calc      114.00, Rep    114.3(4) ......  Senseless s.u.
              C25     -C27     -H27A       1.555  1.555  1.555     #  147 Check
PLAT752_ALERT_4_C Angle   Calc      115.00, Rep    114.6(4) ......  Senseless s.u.
              C25     -C27     -H27B       1.555  1.555  1.555     #  148 Check
PLAT752_ALERT_4_C Angle   Calc      112.00, Rep    112.4(4) ......  Senseless s.u.
              C25     -C27     -H27C       1.555  1.555  1.555     #  149 Check
PLAT752_ALERT_4_C Angle   Calc      105.00, Rep    104.8(3) ......  Senseless s.u.
              H27A    -C27     -H27B       1.555  1.555  1.555     #  150 Check
PLAT752_ALERT_4_C Angle   Calc      105.00, Rep    104.9(3) ......  Senseless s.u.
              H27A    -C27     -H27C       1.555  1.555  1.555     #  151 Check
PLAT752_ALERT_4_C Angle   Calc      105.00, Rep    104.9(3) ......  Senseless s.u.
              H27B    -C27     -H27C       1.555  1.555  1.555     #  152 Check
PLAT752_ALERT_4_C Angle   Calc      114.00, Rep    114.4(4) ......  Senseless s.u.
              C25     -C26     -H26A       1.555  1.555  1.555     #  153 Check
PLAT752_ALERT_4_C Angle   Calc      112.00, Rep    112.2(4) ......  Senseless s.u.
              C25     -C26     -H26B       1.555  1.555  1.555     #  154 Check
PLAT752_ALERT_4_C Angle   Calc      115.00, Rep    115.3(4) ......  Senseless s.u.
              C25     -C26     -H26C       1.555  1.555  1.555     #  155 Check
PLAT752_ALERT_4_C Angle   Calc      105.00, Rep    104.5(3) ......  Senseless s.u.
              H26A    -C26     -H26B       1.555  1.555  1.555     #  156 Check
PLAT752_ALERT_4_C Angle   Calc      105.00, Rep    104.6(3) ......  Senseless s.u.
              H26A    -C26     -H26C       1.555  1.555  1.555     #  157 Check
PLAT752_ALERT_4_C Angle   Calc      105.00, Rep    104.8(3) ......  Senseless s.u.
              H26B    -C26     -H26C       1.555  1.555  1.555     #  158 Check
PLAT911_ALERT_3_C Missing FCF Refl Between Thmin & STh/L=    0.588         40 Report
```

Alert level G

```
PLAT002_ALERT_2_G Number of Distance or Angle Restraints on AtSite         61 Note
PLAT007_ALERT_5_G Number of Unrefined Donor-H Atoms ..............          4 Report
PLAT032_ALERT_4_G Std. Uncertainty on Flack Parameter Value High .      0.400 Report
```

```
PLAT042_ALERT_1_G Calc. and Reported MoietyFormula Strings  Differ     Please Check
PLAT045_ALERT_1_G Calculated and Reported Z Differ by a Factor ...       2.00 Check
PLAT072_ALERT_2_G SHELXL First  Parameter in WGHT  Unusually Large       0.11 Report
PLAT172_ALERT_4_G The CIF-Embedded .res File Contains DFIX Records          4 Report
PLAT176_ALERT_4_G The CIF-Embedded .res File Contains SADI Records          7 Report
PLAT395_ALERT_2_G Deviating  X-O-Y    Angle From 120 for O5             109.1 Degree
PLAT720_ALERT_4_G Number of Unusual/Non-Standard Labels ..........          4 Note
PLAT790_ALERT_4_G Centre of Gravity not Within Unit Cell: Resd.  #          3 Note
              H2 O
PLAT791_ALERT_4_G Model has Chirality at C3         (Sohnke SpGr)           S Verify
PLAT791_ALERT_4_G Model has Chirality at C4         (Sohnke SpGr)           S Verify
PLAT791_ALERT_4_G Model has Chirality at C5         (Sohnke SpGr)           R Verify
PLAT791_ALERT_4_G Model has Chirality at C7         (Sohnke SpGr)           S Verify
PLAT791_ALERT_4_G Model has Chirality at C8         (Sohnke SpGr)           S Verify
PLAT791_ALERT_4_G Model has Chirality at C9         (Sohnke SpGr)           S Verify
PLAT791_ALERT_4_G Model has Chirality at C10        (Sohnke SpGr)           R Verify
PLAT791_ALERT_4_G Model has Chirality at C13        (Sohnke SpGr)           R Verify
PLAT791_ALERT_4_G Model has Chirality at C14        (Sohnke SpGr)           S Verify
PLAT791_ALERT_4_G Model has Chirality at C17        (Sohnke SpGr)           R Verify
PLAT791_ALERT_4_G Model has Chirality at C20        (Sohnke SpGr)           R Verify
PLAT860_ALERT_3_G Number of Least-Squares Restraints .............        393 Note
PLAT883_ALERT_1_G No Info/Value for _atom_sites_solution_primary .     Please Do !
PLAT909_ALERT_3_G Percentage of I>2sig(I) Data at Theta(Max) Still        62% Note
PLAT910_ALERT_3_G Missing # of FCF Reflection(s) Below Theta(Min).          1 Note
PLAT941_ALERT_3_G Average HKL Measurement Multiplicity ...........        3.6 Low
PLAT978_ALERT_2_G Number C-C Bonds with Positive Residual Density.          4 Info
```

---

0 **ALERT level A** = Most likely a serious problem - resolve or explain
3 **ALERT level B** = A potentially serious problem, consider carefully
139 **ALERT level C** = Check. Ensure it is not caused by an omission or oversight
28 **ALERT level G** = General information/check it is not something unexpected

3 ALERT type 1 CIF construction/syntax error, inconsistent or missing data
5 ALERT type 2 Indicator that the structure model may be wrong or deficient
36 ALERT type 3 Indicator that the structure quality may be low
125 ALERT type 4 Improvement, methodology, query or suggestion
1 ALERT type 5 Informative message, check

It is advisable to attempt to resolve as many as possible of the alerts in all categories. Often the minor alerts point to easily fixed oversights, errors and omissions in your CIF or refinement strategy, so attention to these fine details can be worthwhile. In order to resolve some of the more serious problems it may be necessary to carry out additional measurements or structure refinements. However, the purpose of your study may justify the reported deviations and the more serious of these should normally be commented upon in the discussion or experimental section of a paper or in the "special_details" fields of the CIF. checkCIF was carefully designed to identify outliers and unusual parameters, but every test has its limitations and alerts that are not important in a particular case may appear. Conversely, the absence of alerts does not guarantee there are no aspects of the results needing attention. It is up to the individual to critically assess their own results and, if necessary, seek expert advice.

Publication of your CIF in IUCr journals

A basic structural check has been run on your CIF. These basic checks will be run on all CIFs submitted for publication in IUCr journals (*Acta Crystallographica*, *Journal of Applied Crystallography*, *Journal of Synchrotron Radiation*); however, if you intend to submit to *Acta Crystallographica Section C* or *E* or *IUCrData*, you should make sure that full publication checks are run on the final version of your CIF prior to submission.

Publication of your CIF in other journals

Please refer to the *Notes for Authors* of the relevant journal for any special instructions relating to CIF submission.

PLATON version of 18/09/2020; check.def file version of 20/08/2020

Datablock castaneroxy-b - ellipsoid plot

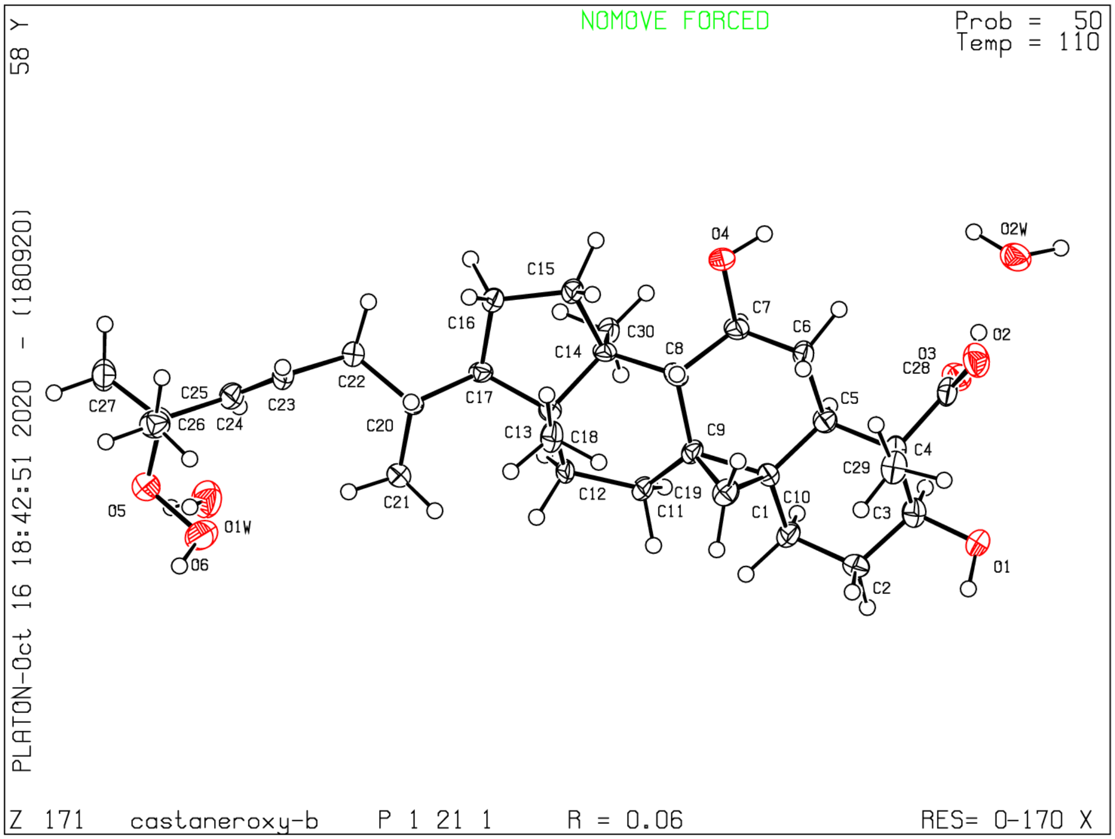

Supplement: Supplementary file 1 [file DataSheet2.PDF]
