# Supplementary material for: Castaneroxy A From the Leaves of Castanea sativa Inhibits Virulence in Staphylococcus aureus
Source: Front Pharmacol. 2021 Jun 28;12:640179. doi: 10.3389/fphar.2021.640179 (PMC8274328; doi:10.3389/fphar.2021.640179)

checkCIF/PLATON report

Structure factors have been supplied for datablock(s) 224C-F2c-PF42-SF7_nsphera

THIS REPORT IS FOR GUIDANCE ONLY. IF USED AS PART OF A REVIEW PROCEDURE FOR PUBLICATION, IT SHOULD NOT REPLACE THE EXPERTISE OF AN EXPERIENCED CRYSTALLOGRAPHIC REFEREE.

No syntax errors found. CIF dictionary Interpreting this report

Datablock: 224C-F2c-PF42-SF7_nsphera

```
Bond precision:  C-C = 0.0012 A          Wavelength=0.71073

Cell:           a=7.54880(14)    b=11.2160(2)         c=16.9599(3)
                alpha=90         beta=102.5498(18)    gamma=90
Temperature:    100 K

                Calculated                 Reported
Volume          1401.64(4)                 1401.64(5)
Space group     P 21                       P 1 21 1
Hall group      P 2yb                      P 2yb
Moiety formula  C30 H48 O6, H2 O           C30 H48 O6, H2 O
Sum formula     C30 H50 O7                 C30 H50 O7
Mr              522.70                     522.73
Dx,g cm-3       1.238                      1.239
Z               2                          2
Mu (mm-1)       0.086                      0.086
F000            572.0                      572.2
F000'           572.28
h,k,lmax        13,19,29                   12,19,29
Nref            15288[ 7939]               12725
Tmin,Tmax       0.988,0.995                0.553,1.000
Tmin'           0.985

Correction method= # Reported T Limits: Tmin=0.553 Tmax=1.000
AbsCorr = MULTI-SCAN

Data completeness= 1.60/0.83      Theta(max)= 38.010

R(reflections)= 0.0377( 11422)    wR2(reflections)= 0.0953( 12725)

S = 1.051                  Npar= 779
```

The following ALERTS were generated. Each ALERT has the format
test-name_ALERT_alert-type_alert-level.
Click on the hyperlinks for more details of the test.

Alert level A

```
PLAT355_ALERT_3_A Long   O-H (X0.82,N0.98A)  O6       - H6       .       1.15 Ang.
```

Author Response: This is a real experimentally observed distance involving a peroxo hydrogen. This H donates a strong hydrogen bond to a water.

Alert level B

```
PLAT417_ALERT_2_B Short Inter D-H..H-D       H1       ..H1WB     .       2.03 Ang.
                                              -1+x,y,1+z  =      1_456 Check
```

Author Response: This is a real experimentally observed distance, with one hydrogen being a strong hydrogen bond donor.

```
PLAT417_ALERT_2_B Short Inter D-H..H-D       H1       ..H3       .       2.09 Ang.
                                            -x,1/2+y,2-z  =      2_557 Check
```

Author Response: This is a real experimentally observed distance, with one hydrogen being a strong hydrogen bond donor.

```
PLAT926_ALERT_1_B Reported and Calculated   R1 Differ by .........    -0.0130 Check
```

Author Response: Refinement using NoSpherA2, an implementation of NOn-SPHERical Atom-form-factors in Olex2. The calculated R1 is based on spherical form factors.

```
PLAT927_ALERT_1_B Reported and Calculated  wR2 Differ by .........    -0.0363 Check
```

Author Response: Refinement using NoSpherA2, an implementation of NOn-SPHERical Atom-form-factors in Olex2. The calculated wR2 is based on spherical form factors.

```
PLAT928_ALERT_1_B Reported and Calculated    S value   Differ by .     -0.400 Check
```

Author Response: Refinement using NoSpherA2, an implementation of NOn-SPHERical Atom-form-factors in Olex2. The calculated S is based on spherical form factors.

Alert level C

```
PLAT222_ALERT_3_C NonSolvent Resd 1  H   Uiso(max)/Uiso(min) Range        5.4 Ratio
PLAT351_ALERT_3_C Long   C-H (X0.96,N1.08A)  C7       - H7       .       1.11 Ang.
PLAT351_ALERT_3_C Long   C-H (X0.96,N1.08A)  C8       - H8A      .       1.11 Ang.
PLAT351_ALERT_3_C Long   C-H (X0.96,N1.08A)  C16      - H16A     .       1.15 Ang.
PLAT351_ALERT_3_C Long   C-H (X0.96,N1.08A)  C18      - H18      .       1.11 Ang.
PLAT351_ALERT_3_C Long   C-H (X0.96,N1.08A)  C25      - H25B     .       1.12 Ang.
PLAT351_ALERT_3_C Long   C-H (X0.96,N1.08A)  C26      - H26B     .       1.11 Ang.
PLAT351_ALERT_3_C Long   C-H (X0.96,N1.08A)  C27      - H27      .       1.12 Ang.
PLAT351_ALERT_3_C Long   C-H (X0.96,N1.08A)  C29      - H29C     .       1.15 Ang.
PLAT417_ALERT_2_C Short Inter D-H..H-D       H1WB     ..H6       .       2.14 Ang.
                                                      x,y,z  =      1_555 Check
```

Author Response: This is a real experimentally observed distance, with one hydrogen being a strong hydrogen bond donor.

```
PLAT911_ALERT_3_C Missing FCF Refl Between Thmin & STh/L=    0.600          8 Report
PLAT915_ALERT_3_C No Flack x Check Done: Low Friedel Pair Coverage         71 %
PLAT975_ALERT_2_C Check Calcd Resid. Dens.  0.60A   From O1              0.41 eA-3
PLAT976_ALERT_2_C Check Calcd Resid. Dens.  0.48A   From O6             -0.45 eA-3
```

Alert level G

```
PLAT002_ALERT_2_G Number of Distance or Angle Restraints on AtSite          6 Note
PLAT003_ALERT_2_G Number of Uiso or Uij Restrained non-H Atoms ...         37 Report
PLAT068_ALERT_1_G Reported F000 Differs from Calcd (or Missing)...     Please Check
PLAT172_ALERT_4_G The CIF-Embedded .res File Contains DFIX Records          1 Report
PLAT173_ALERT_4_G The CIF-Embedded .res File Contains DANG Records          1 Report
PLAT176_ALERT_4_G The CIF-Embedded .res File Contains SADI Records          1 Report
PLAT186_ALERT_4_G The CIF-Embedded .res File Contains ISOR Records          2 Report
PLAT187_ALERT_4_G The CIF-Embedded .res File Contains RIGU Records          2 Report
PLAT395_ALERT_2_G Deviating  X-O-Y    Angle From 120 for O5             107.7 Degree
PLAT720_ALERT_4_G Number of Unusual/Non-Standard Labels ..........          2 Note
PLAT769_ALERT_4_G CIF Embedded explicitly supplied scattering data     Please Note
PLAT790_ALERT_4_G Centre of Gravity not Within Unit Cell: Resd.  #          2 Note
              H2 O
PLAT791_ALERT_4_G Model has Chirality at C1          (Sohnke SpGr)          S Verify
PLAT791_ALERT_4_G Model has Chirality at C2          (Sohnke SpGr)          S Verify
PLAT791_ALERT_4_G Model has Chirality at C3          (Sohnke SpGr)          R Verify
PLAT791_ALERT_4_G Model has Chirality at C4          (Sohnke SpGr)          R Verify
PLAT791_ALERT_4_G Model has Chirality at C5          (Sohnke SpGr)          S Verify
PLAT791_ALERT_4_G Model has Chirality at C6          (Sohnke SpGr)          S Verify
PLAT791_ALERT_4_G Model has Chirality at C7          (Sohnke SpGr)          S Verify
PLAT791_ALERT_4_G Model has Chirality at C14         (Sohnke SpGr)          R Verify
PLAT791_ALERT_4_G Model has Chirality at C15         (Sohnke SpGr)          S Verify
PLAT791_ALERT_4_G Model has Chirality at C18         (Sohnke SpGr)          R Verify
PLAT791_ALERT_4_G Model has Chirality at C23         (Sohnke SpGr)          R Verify
PLAT791_ALERT_4_G Model has Chirality at C27         (Sohnke SpGr)          R Verify
PLAT802_ALERT_4_G CIF Input Record(s) with more than 80 Characters          1 Info
PLAT860_ALERT_3_G Number of Least-Squares Restraints .............        792 Note
PLAT883_ALERT_1_G No Info/Value for _atom_sites_solution_primary .     Please Do !
PLAT910_ALERT_3_G Missing # of FCF Reflection(s) Below Theta(Min).          1 Note
PLAT912_ALERT_4_G Missing # of FCF Reflections Above STh/L=  0.600        391 Note
PLAT916_ALERT_2_G Hooft y and Flack x Parameter Values Differ by .       0.41 Check
PLAT933_ALERT_2_G Number of OMIT Records in Embedded .res File ...         13 Note
PLAT941_ALERT_3_G Average HKL Measurement Multiplicity ...........        4.0 Low
PLAT978_ALERT_2_G Number C-C Bonds with Positive Residual Density.         11 Info
PLAT982_ALERT_1_G The  C-f′=    0.0021 Deviates from  IT-value =       0.0033 Check
```

```
PLAT982_ALERT_1_G The  O-f'=    0.0079 Deviates from  IT-value =       0.0106 Check
```

```
  1 ALERT level A = Most likely a serious problem - resolve or explain
  5 ALERT level B = A potentially serious problem, consider carefully
 14 ALERT level C = Check. Ensure it is not caused by an omission or oversight
 35 ALERT level G = General information/check it is not something unexpected

  7 ALERT type 1 CIF construction/syntax error, inconsistent or missing data
 11 ALERT type 2 Indicator that the structure model may be wrong or deficient
 15 ALERT type 3 Indicator that the structure quality may be low
 22 ALERT type 4 Improvement, methodology, query or suggestion
  0 ALERT type 5 Informative message, check
```

It is advisable to attempt to resolve as many as possible of the alerts in all categories. Often the minor alerts point to easily fixed oversights, errors and omissions in your CIF or refinement strategy, so attention to these fine details can be worthwhile. In order to resolve some of the more serious problems it may be necessary to carry out additional measurements or structure refinements. However, the purpose of your study may justify the reported deviations and the more serious of these should normally be commented upon in the discussion or experimental section of a paper or in the "special_details" fields of the CIF. checkCIF was carefully designed to identify outliers and unusual parameters, but every test has its limitations and alerts that are not important in a particular case may appear. Conversely, the absence of alerts does not guarantee there are no aspects of the results needing attention. It is up to the individual to critically assess their own results and, if necessary, seek expert advice.

Publication of your CIF in IUCr journals

A basic structural check has been run on your CIF. These basic checks will be run on all CIFs submitted for publication in IUCr journals (*Acta Crystallographica*, *Journal of Applied Crystallography*, *Journal of Synchrotron Radiation*); however, if you intend to submit to *Acta Crystallographica Section C* or *E* or *IUCrData*, you should make sure that full publication checks are run on the final version of your CIF prior to submission.

Publication of your CIF in other journals

Please refer to the *Notes for Authors* of the relevant journal for any special instructions relating to CIF submission.

PLATON version of 18/09/2020; check.def file version of 20/08/2020

Datablock 224C-F2c-PF42-SF7_nsphera - ellipsoid plot

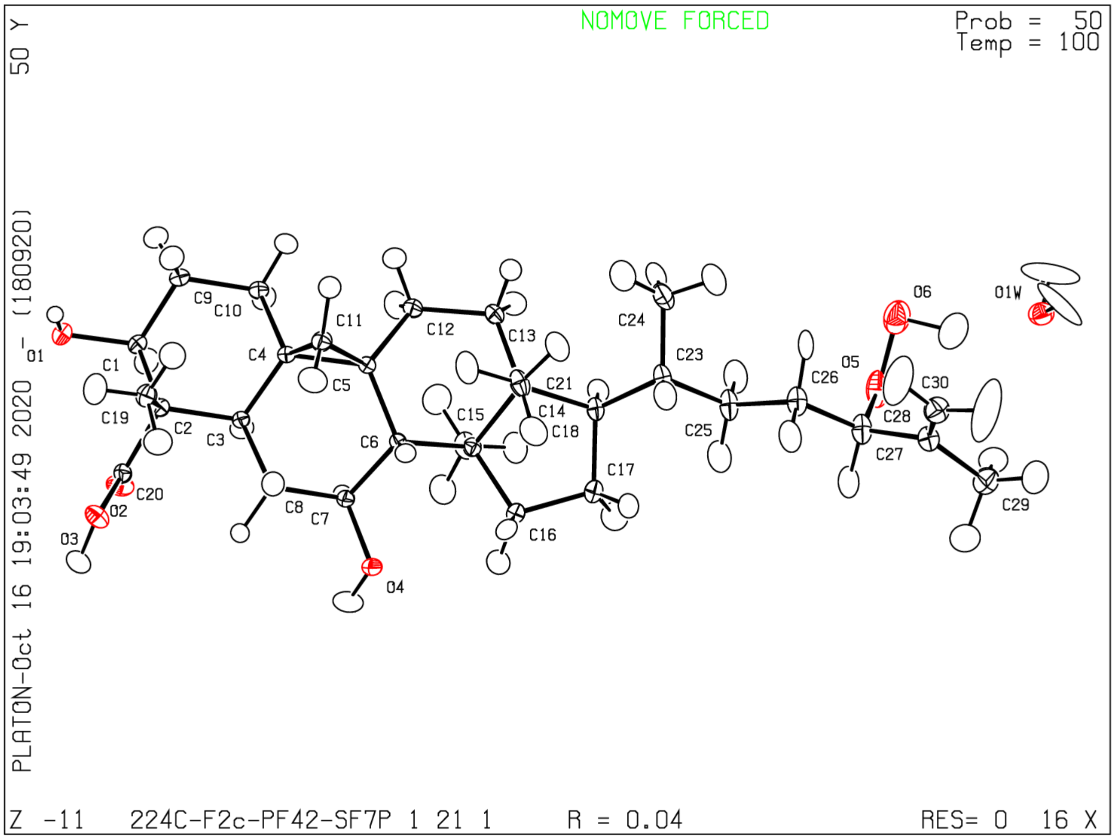

Supplement: Supplementary file 3 [file DataSheet1.PDF]
